# Using Friends as Sensors to Detect Global-Scale Contagious Outbreaks

**DOI:** 10.1371/journal.pone.0092413

**Published:** 2014-04-09

**Authors:** Manuel Garcia-Herranz, Esteban Moro, Manuel Cebrian, Nicholas A. Christakis, James H. Fowler

**Affiliations:** 1 Department of Computer Science, Escuela Politécnica Superior, Universidad Autónoma de Madrid, Madrid, Spain; 2 Department of Mathematics & GISC, Universidad Carlos III de Madrid, Leganés, Spain; 3 Instituto de Ingeniería del Conocimiento, Universidad Autónoma de Madrid, Madrid, Spain; 4 Computer Science & Engineering Department, University of California San Diego, San Diego, California, United States of America; 5 Media Laboratory, Massachusetts Institute of Technology, Cambridge, Massachusetts, United States of America; 6 National Information and Communications Technology Australia, Melbourne, Victoria, Australia; 7 Department of Sociology, Yale University, New Haven, Connecticut, United States of America; 8 Department of Ecology and Evolutionary Biology, Yale University, New Haven, Connecticut, United States of America; 9 Department of Medicine, Yale School of Medicine, New Haven, Connecticut, United States of America; 10 Medical Genetics Division, School of Medicine, University of California San Diego, San Diego, California, United States of America; 11 Political Science Department, University of California San Diego, San Diego, California, United States of America; Instituto de Fisica Interdisciplinar y Sistemas Complejos IFISC (CSIC-UIB), Spain

## Abstract

Recent research has focused on the monitoring of global–scale online data for improved detection of epidemics, mood patterns, movements in the stock market political revolutions, box-office revenues, consumer behaviour and many other important phenomena. However, privacy considerations and the sheer scale of data available online are quickly making global monitoring infeasible, and existing methods do not take full advantage of local network structure to identify key nodes for monitoring. Here, we develop a model of the contagious spread of information in a global-scale, publicly-articulated social network and show that a simple method can yield not just early detection, but advance warning of contagious outbreaks. In this method, we randomly choose a small fraction of nodes in the network and then we randomly choose a friend of each node to include in a group for local monitoring. Using six months of data from most of the full Twittersphere, we show that this friend group is more central in the network and it helps us to detect viral outbreaks of the use of novel hashtags about 7 days earlier than we could with an equal-sized randomly chosen group. Moreover, the method actually works better than expected due to network structure alone because highly central actors are both more active and exhibit increased diversity in the information they transmit to others. These results suggest that local monitoring is not just more efficient, but also more effective, and it may be applied to monitor contagious processes in global–scale networks.

## Introduction

Modern social, informational, and transactional platforms offer a means for information to spread naturally (e.g, as in the case of the “Arab Spring” [1]), and there is increasing interest in using these systems to intentionally promote the spread of information and behavior [2]–[5]. In addition, they also yield a brand-new and large-scale global view of social interactions and dynamics of formerly hidden phenomena [6]. Recent work has taken advantage of such monitoring of global-scale online data for improved detection of epidemics [7]–[10], mood patterns [11], [12], stock performance [13], political revolutions [14], box-office revenues [15], consumer behavior [9], [16] and many other important phenomena. However, the advent of global monitoring has recently heightened concerns about privacy [17], and anonymization is often insufficient to guarantee it [18]. Thus, future efforts to monitor global phenomena may be restricted to analysis at a local scale [10], [19] or to incomplete pictures of the system. Moreover, the explosive growth of online data has made it more and more difficult to perform a complete global analysis. As a result, scholars are beginning to develop local methods that sample small but relevant parts of the system [20], [21].

Here, we elaborate the theoretical framework of [22] sampling technique to take advantage of the local structure inherent in large-scale online social networks, to allow monitoring of a network without relying on a complete picture of the system; and we use it to test an important hypothesis about non–biological social contagion.

If a message is transmitted exogenously via *broadcast*, then all individuals are equally likely to receive it, regardless of their position in the network. On the other hand, if a message is transmitted endogenously from person to person to person via *contagion*, then individuals at the center of a network are likely to receive it sooner than randomly-chosen members of the population because central individuals are a smaller number of steps (degrees of separation) away from the average individual in the network [22], [23]. As a result, for contagious processes, we would expect the S-shaped cumulative “epidemic curve” [24] to be shifted to the left (forward in time) for centrally located individuals compared to the population as a whole.

If so, then the careful collection of information from a sample of central individuals within human social networks could be used to detect contagious outbreaks before they happen in the population at large [22]. We call this the *sensor hypothesis*. In fact, the very discrepancy in the time to infection between central and randomly-chosen individuals could serve as a means to distinguish between exogenous and endogenous mechanisms, either *ex post* by comparing their mean times of infection or in real time by looking for the first day in which there is a significant divergence in their cumulative incidences.

## Results

Using 6 months of data from Twitter recorded in 2009 [25], we analyze a network containing 40 million users around the world who are connected by 1.5 billion directed relationships (“follows”). Over six months, these users sent nearly half a billion messages (“tweets”), of which 67 million contained a user-supplied topic keyword called a “hashtag”. These hashtags are prefixed by a pound sign (#) and are used to denote unique people, events, or ideas, making them useful for studying the spread of information online [26]–[28].

To test the sensor hypothesis, we need a sample of individuals with higher network centrality (the “sensor” group) to compare with a sample of randomly chosen individuals (the “control” group). However, measuring centrality can be a computationally expensive task in large-scale networks like Twitter (see SI). Therefore, we use a simplified approach that first randomly selects a set of users for the control group, and then randomly chooses “friends” of members of this group to put in an equally-sized sensor group. This procedure generates a sensor group with higher degree centrality than the control group because of the “friendship paradox”: high-degree individuals are more likely to be connected to a randomly chosen person than low-degree individuals [22], [29]. In other words, “your friends have more friends than you do” [30].

**Figure 1:**
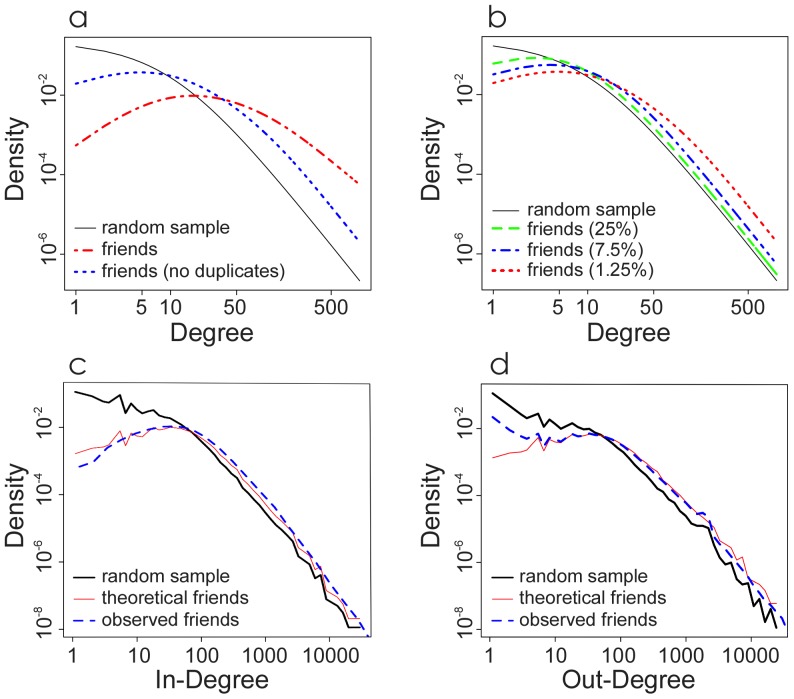
Twitter exhibits the “friendship paradox”. a) Expected degree distributions for a 1.25% random sample of the Twitter network (black line), friends of this randomly chosen group (red line), and the same friends group with duplicates removed (blue line); b) Larger samples of friends show a smaller difference in degree distribution from the overall network (black = overall network, green = 25% sample, blue = 7.5% sample, red = 1.25%); c) and d) Respectively, In-degree (follower) and out-degree (followee) distribution of a random sample of 500,000 users, 1.25% of Twitters users (the “control” group, black line) and the theoretical (red line) and observed (blue line) in-degree and out-degree distributions of their friends (the “sensor” group) with duplicates from the friends group removed.

In Fig. 1a we demonstrate that the sensor group contains more high degree individuals and fewer low degree individuals, and this is true even if we remove duplicates from the sensor group (duplicates occur when the same person is randomly chosen as a friend by multiple individuals in the control group). However, this difference between the sensor and control groups depends on what fraction of the network is sampled. As the fraction increases, there is increasing overlap between the two groups, reducing the difference in their degree distributions (Fig. 1b). We derive closed form equations that characterize the expected degree distribution for both the sensor groups (with and without duplicates) and control groups based on the fraction of nodes sampled and an arbitrary known degree distribution for the network as a whole (see SI “An Analytic Elaboration of the Friendship Paradox”). Fig. 1c,d show that these equations fit the data well for a random sample of 1.25% of all users (500,000 total) on Twitter, confirming our expectation that the sensor group is more central than the control group.

To test whether sensors can provide early warning of a contagious message spreading through the network, suppose 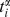 denotes the time at which a sampled user 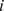 first mentions hashtag 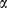 (i.e the infection time). We would expect 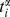 to be smaller on average for users belonging to a central sensor group 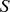 than for those of a random control group 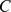. If we denote 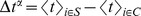 for hashtag 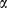, the sensor hypothesis is that 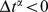.

**Figure 2:**
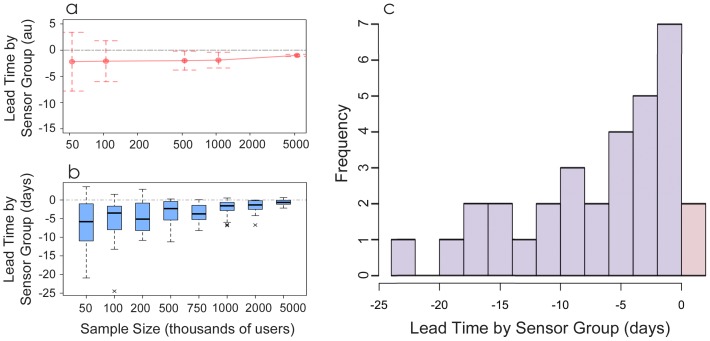
Friends as sensors yield early detection of the use of hashtags. a) Measures of lead times based on simulations of an infection spreading through a network with infection probability 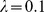 and recovery probability 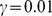 on a Barabasi-Albert random network with tail exponent 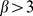 show that a sensor group tends to provide earlier warning than a randomly-chosen control group in smaller samples, but decreasing sampling variation in larger sample sizes means that the statistical likelihood of providing early warning is maximized in moderately-sized samples. b) Observed results for hashtags on Twitter used by 1% of the individuals using a hashtag of each sample. c) Average lead time of first usage of each hashtag in the sensor group vs. the control group for all hashtags used by at least 10 users in each of 5 random samples of 50,000 random users.

However, note that 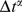 depends on the size of the samples in two ways. For small samples, the number of “infected” users (i.e. users mentioning hashtag 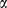) will be scarce, leading to large statistical errors. On the other hand, for big samples, the degree distribution of the control and sensor groups tend to overlap and consequently 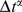 approaches 0. Therefore, it may be necessary to find an optimal “Goldilocks” sample size that gives statistical power while still preserving the high-centrality characteristic of the sensor group. Fig. 2a shows results from a theoretical simulation of an infection [31] spreading in a synthetic network (see SI “Sensor Performance in a Simulated Infection Model”) while Fig. 2b shows an empirical analysis of widely used hashtags in our Twitter database (see SI “Sensor Performance in Real Data”). Both theory and data suggest that there exists an optimal (and moderate) sample size that may perform best for detecting large and significant differences between the sensor and control group resulting from contagious processes.

To analyze the performance of the sensor mechanism, we collected five random control samples of 50,000 users and a random set of their followees of the same size to use as sensors for each one. Focusing on the 32 most widespread hashtags that appear at least 10 times in each control sample, Fig. 2c shows that 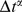 is negative (i.e., the sensor sample uses the hashtag prior to the control sample) in all but two cases, with a mean for all hashtags of 7.1 days (SEM 1.1 days). In the SI “Using the Sensor Method with a Small Set of Samples”, we also show this distribution for a wider range of hashtags, and these all show that 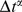 tends to be negative. In other words, the sensor groups provide advance warning of the usage of a wide variety of hashtags.

**Figure 3:**
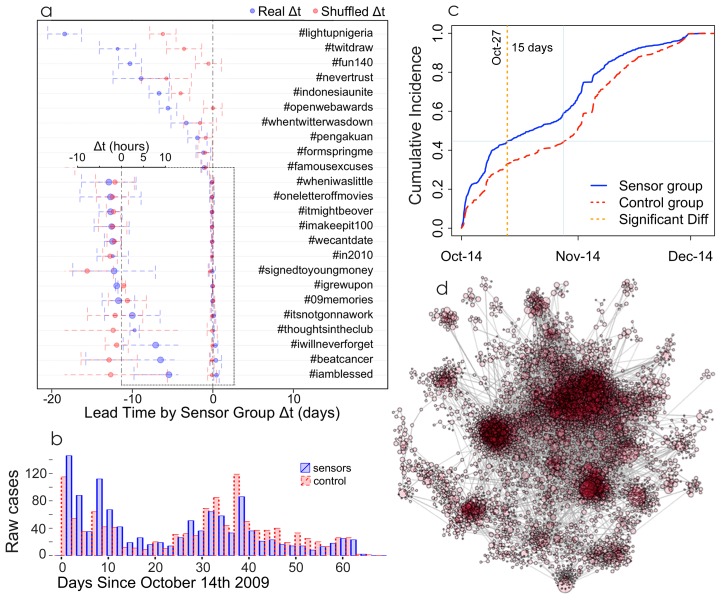
Signs of virality in hashtag usage. a) The average lead for the 24 most-used hashtags time across 1,000 trials of the sensor group (in blue) vs. the same calculated lead time when all times of hashtag usage are randomly shuffled (in red). Vertical bars are SEM.; b) daily incidence and c) cumulative daily incidence for the hashtag #openwebawards show a shift forward in the S-shaped epidemic curve and a burst in the sensor group relative to the control group that could be used to predict the outbreak of this hashtag on the 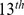 day (the first day on which, using all available information up to that day, there is a significant difference between the sensor and control groups with p-value
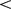
0.05), 15 days before the control group reaches the same cumulative incidence and before the estimated peak in daily incidence; d) greatest connected component of the follower network of users using the #openwebawards hashtag shows that many users are connected in a large component.

We also hypothesized that comparative monitoring of a sensor group and a control group may help distinguish which hashtags are spreading virally via a contagious process and which are spreading via broadcast. We studied 24 hashtags (Fig. 3a) that were “born” during our sample period (they first appeared at least 25 days after the start date of data collection) and then became widely used (they were eventually used more than 20,000 times). Notably, the users using these hashtags tended to be highly connected and many were connected to a giant component, a sign that the hashtags may have spread virally online from user to user (see Fig. 3d and Fig. S11 to S14 in File S1 for more examples).

For each of these hashtag networks, we constructed a random control sample of 5% its size and a similarly-sized sensor sample of their followees to calculate 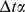. We then repeated this process 1,000 times to generate a statistical distribution of these observed lead times (as in Fig. 2c). The sensor group led the control group (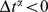) 79.9% (SE 1.2%) of the time. However, note that there was considerable variation in lead times, from 20 days to a few hours or no advance warning.

An alternative explanation to the sensors lead time might be that hashtags are more likely to be created by the most active users such as the ones in the sensor group, and that, being more central, they are in a better position to make them popular; or from the opposite perspective, that sensors end up being more central because they create content that end up trending. In other words, that central actors select novel topics rather than being agents of contagion. In order to evaluate this possibility, we calculated the exposure rates of sensors and controls (i.e. the number of users who used the hashtag after being exposed to it). The results (see SI “Using the Sensor Method with Hashtag Networks”) show that the exposure rate is significantly higher in the sensor group, meaning that sensors are better transmitters in Twitter (they are aware of whats happening in Twitter and transmit it very soon) while controls seem to introduce more information in Twitter from other sources (or to create it), rather than transmitting what they are exposed to in Twitter. These findings therefore militate against the selection idea in favor of the contagion hypothesis.

To see how the sensor method works for hashtags that are *not* spreading virally, we generated a null distribution in which we randomly shuffled the timestamp of each hashtag use within the fully observed data, and then measured the resulting difference in the sensor and control group samples, 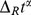. There is a positive correlation between degree and number of tweets per day so, having higher degrees on average than controls, sensors also tend to tweet more often. Therefore, in the shuffling process sensors actually have a greater chance of getting smaller times of infection than controls because they have more tweets to be assigned a new timestamp. By shuffling the timestamps of every tweet we are measuring the lead time sensors would get not because of their centrality in a viral process but because of their higher tweeting rates. The difference, therefore, between this lead time and the observed one corresponds to the viral component of the process. Again, we repeated the procedure 1,000 times to generate a statistical distribution (see SI “Using the Sensor Method with Hashtag Networks”). The results show that the observed distribution of lead times falls outside the null distribution for 65.4% (SE 1.2%) of the hashtags, suggesting they did, in fact, spread virally (Fig. 3a).

The hashtags also generally showed a shift forward in the daily and cumulative incidence curves of the sensor group compared to the control one (Fig. 3c,d). This shift forward, another sign of virality in itself, could allow for identification of an outbreak in advance, as the sensors deviation from the trajectory of the control group identifies a process that is spreading through the network, affecting central individuals faster than random ones. For example, estimating the models each day using all available information up to that day, for #openwebawards users, we find two consecutive days of significant (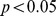) lead time by the sensor group compared to the control group on day 13, a full 15 days before the estimated peak in daily incidence (see SI “Using the Sensor Method with Hashtag Networks” and Fig. S11 to S14 in File S1), and also 15 full days before the control sample reaches the same incidence as the sensor group (See Fig. 3c).

**Figure 4:**
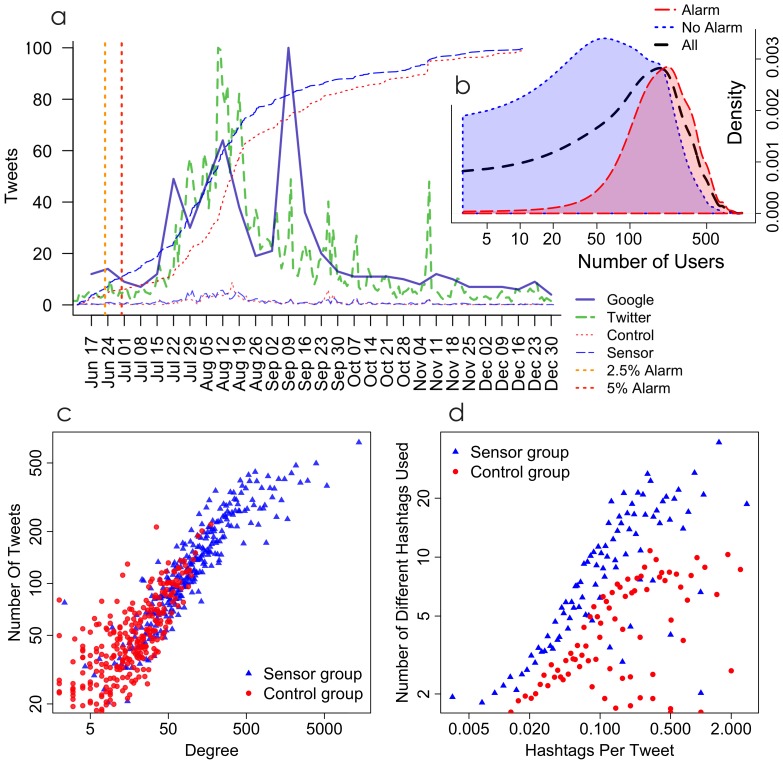
Early warnings of the sensor mechanism and differences between users in the sensor and control groups. a) The Twitter sensor sample anticipates outbreaks in both Twitter hashtags and Google searches. The purple solid line shows a normalized measure of the number of Google searches per day for “health care”. The green dashed line shows the a normalized measure of the number of tweets using the hashtag #healthcare per day. Thinner lines at the bottom show normalized daily incidence (DI) for the control (dotted red) and sensor (dashed blue) groups. Thinner lines from the bottom left to the upper right show the empirical cumulative distribution (ECDF) of control (dotted red) and sensor (dashed blue) groups. Vertical dotted lines show dates when an alarm was first triggered by a 2.5% divergence (orange) and 5% divergence (red) in the sensor and control groups. b) An early warning alarm triggered by a 0.25% divergence in the sensor and control groups predicts overall usage with relatively few false positives (see SI “Reproduction Rates of Hashtags as a Factor Affecting Early Detection” for details). c & d) Users in the sensor group (blue) are more active (c) and also use a wider variety of hashtags (d) than those in the control group (red), even controlling for activity. These attributes both contribute to early warning provided by the sensor groups structural position.

One can also use fixed thresholds to trigger a “divergence alarm” when the sensor group usage of a particular hashtag is growing faster than the control group usage. We tested a variety of these thresholds (see SI “Reproduction Rates of Hashtags as a Factor Affecting Early Detection”) and found that they consistently provided advance warning of the hashtags that would be most likely to yield high usage in future. In Fig. 4a, we show that the false positive rate for these alarms (an alarm that was triggered by a hashtag that would not be widely used) is low. In Fig. 4b, we also show that the alarms can anticipate behavior outside Twitter as well. A survey of several Google search terms that are closely related to certain hashtags in our data shows that the peaks in Twitter usage tend to precede or coincide with Google Trends peaks, and thus increases in the Twitter sensor group and their divergence with the control group provide early warning not only on Twitter but on Google searches as well (see SI “Twitter, Sensors in Twitter, and Google Trends” for several examples).

Finally, while the sensor mechanism allows us to identify a more central group, in terms of degree–centrality, that can be used to detect contagious outbreaks in advance, it may also allow us to focus on users who have other characteristics that could improve monitoring. First, in terms of network centrality, we have found sensors to have also greater betweenness. Second, in terms of activity, users in the sensor group may be more central because they are more active on twitter, and indeed we find this to be true too (Fig. 4c). On average, users in the sensor group sent 154 tweets (SE 2.8) during the six months they were monitored, while users in the control group tweeted only 55 times (SE 1.0, difference of means t = 36, 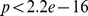). However, we also find that sensor users tend to use a greater variety of hashtags, even controlling for activity levels (Fig. 4d) (see SI “Differences in Sensor and Control Characteristics That Also Affect Propagation”). In summary, the sensor mechanism, while targeting users with higher degree centrality, is able to identify users that are more central in many ways.

The distribution of the number of users using any one hashtag is heavy tailed (see SI “The Twitter Data”) with most hashtags being used by less than a few hundred people and very few reaching the tens of thousands. Therefore, for most hashtags, the probability of finding sufficient users to perform a significant analysis in a random sample of Twitter is very small. Yet, despite the relatively small size of the infected populations, the sensor mechanism we test here seems to anticipate the global spread of information in a wide variety of cases. And, importantly, it only requires a tiny fraction of the network as a whole to be monitored, allowing us to find a sample 6 times more connected than selecting the most connected users of a sample 5 times larger (see SI “Friends vs. Most Connected Nodes and Most Connected Friends as Sensors”).

## Discussion

We believe that this method could be applied in a wide variety of contexts in which scholars, policy-makers, and companies are attempting to use “big data” online to predict important phenomena. For example, the sensor method could be used in conjunction with online search to improve surveillance for potential flu outbreaks [8], [22]. By following the online behavior of a group known to be central in a network (for example, based on e-mail records which could be used to construct a friend sensor group), Google or other companies that monitor flu-related search terms might be able to get high-quality, real-time information about a real-world epidemic with greater lead time, giving public health officials even more time to plan a response. Similarly, policy-makers could monitor global mood patterns [12] to anticipate important changes in public sentiment that may influence economic growth, elections, opposition movements, or even political revolutions [14]. We also conjecture that investors might use these methods to better predict movements in the stock market [13].

Just as we find variation in lead time for different hashtags, we expect that the ability of the sensor method to detect outbreaks early, and how early it might do so, will depend on a number of factors, including: the online context (e.g., whether twitter or some other data environment); the intrinsic properties of the phenomenon that is spreading and how it is measured; the size or composition of the population, including the overall prevalence of susceptible or affected individuals; the number of people in the sensor group; the topology of the network (for example, the degree distribution and its variance, or other structural attributes) [23]; and other factors, such as whether the outbreak modifies the structure of the network as it spreads (for example, by affecting the tendency of any two individuals to remain connected after the information is transmitted). Nevertheless, it seems clear that taking advantage of the topological architecture of human populations offers the prospect of detecting a wide variety of contagious informational or behavioral outbreaks in advance of their striking the general population.

## Supporting Information

File S1 Contains the following files: 1 An Analytic Elaboration of the Friendship Paradox (2). 2 The Twitter Data (4). 3 Sensor Performance in a Simulated Infection Model (5). 4 Sensor Performance in Real Data (7). 5 Using the Sensor Method with a Small Set of Samples (8). 6 Using the Sensor Method with Hashtag Networks (9). 7 Reproduction Rates of Hashtags as a Factor Affecting Early Detection (11). 8 Twitter, Sensors in Twitter, and Google Trends (12). 9 Friends vs. Most Connected Nodes and Most Connected Friends as Sensors (14). 10 Differences in Sensor and Control Characteristics That Also Affect Propagation (15). 11 Figures (16): S1 The friendship paradoxes (17). S2 Twitter data overview (18). S3 Hashtags first appearance and popularity (19). S4 Most used hashtags, users and Greatest Connected Component size (20). S5 Variations of lead time of using friends as sensors with sample size for hashtags used by more than 0.01% of all users (21). S6 Variations of lead time of using friends as sensors with sample size for hashtags used by more than 0.04% of all users (22). S7 Variations of lead time of using friends as sensors with sample size for hashtags related to important events (23). S8 Variations of lead time of using friends as sensors with sample size for different hashtags (24). S9 Variations of lead time of using friends as sensors with sample size for hashtags for which the method works (25). S10 Variations of lead time of using friends as sensors with sample size for hashtags for which the method does not work (26). S11 Networks and cumulative distributions for hashtags with the biggest lead times (27). S12 Networks and cumulative distributions for hashtags with big lead times (28). S13 Networks and cumulative distributions for more hashtags with big lead times (29). S14 Networks and cumulative distributions for hashtags with small lead times (30). S15 Variations of sensor lead time with final number of hashtag users (31). S16 Exposure rate of sensor and control populations (32). S17 Variations of sensor lead time and divergence alarms with final number of hashtag users (33). S18 Distribution of number of hashtag users for hashtags that trigger a divergence alarm (34). S19 Distribution of number of users for hashtags triggering a divergence alarm vs. not triggering an alarm (35). S20 Twitter hashtags and Using Friends as Sensors vs. Google searches (36). S20 Continued. Twitter hashtags and Using Friends as Sensors vs. Google searches (37). S20 Continued. Twitter hashtags and Using Friends as Sensors vs. Google searches (38). S21 Lead time of using friends as sensors vs. using sensors by degree (39). S21 Continued. Lead time of using friends as sensors vs. using sensors by degree (40). S21 Continued. Lead time of using friends as sensors vs. using sensors by degree (41). S21 Continued. Lead time of using friends as sensors vs. using sensors by degree (42). S21 Continued. Lead time of using friends as sensors vs. using sensors by degree (43). S21 Continued. Lead time of using friends as sensors vs. using sensors by degree (44) S22 Degree and betweenness differences between controls and sensors (45).
(PDF)
