# Supplementary material for: Using Friends as Sensors to Detect Global-Scale Contagious Outbreaks
Source: PLoS One. 2014 Apr 9;9(4):e92413. doi: 10.1371/journal.pone.0092413 (PMC3981694; doi:10.1371/journal.pone.0092413)
Supplement: File S1 — Contains the following files: 1 An Analytic Elaboration of the Friendship Paradox (2). 2 The Twitter Data (4). 3 Sensor Performance in a Simulated Infection Model (5). 4 Sensor Performance in Real Data (7). 5 Using the Sensor Method with a Small Set of Samples (8). 6 Using the Sensor Method with Hashtag Networks (9). 7 Reproduction Rates of Hashtags as a Factor Affecting Early Detection (11). 8 Twitter, Sensors in Twitter, and Google Trends (12). 9 Friends vs. Most Connected Nodes and Most Connected Friends as Sensors (14). 10 Differences in Sensor and Control Characteristics That Also Affect Propagation (15). 11 Figures (16): S1 The friendship paradoxes (17). S2 Twitter data overview (18). S3 Hashtags first appearance and popularity (19). S4 Most used hashtags, users and Greatest Connected Component size (20). S5 Variations of lead time of using friends as sensors with sample size for hashtags used by more than 0.01% of all users (21). S6 Variations of lead time of using friends as sensors with sample size for hashtags used by more than 0.04% of all users (22). S7 Variations of lead time of using friends as sensors with sample size for hashtags related to important events (23). S8 Variations of lead time of using friends as sensors with sample size for different hashtags (24). S9 Variations of lead time of using friends as sensors with sample size for hashtags for which the method works (25). S10 Variations of lead time of using friends as sensors with sample size for hashtags for which the method does not work (26). S11 Networks and cumulative distributions for hashtags with the biggest lead times (27). S12 Networks and cumulative distributions for hashtags with big lead times (28). S13 Networks and cumulative distributions for more hashtags with big lead times (29). S14 Networks and cumulative distributions for hashtags with small lead times (30). S15 Variations of sensor lead time with final number of hashtag users (31). S16 Exposure rate of sensor and control p [file pone.0092413.s001.pdf]

Supporting Information for

Using friends as sensors to detect global-scale contagious outbreaks

Manuel Garcia-Herranz[1,*], Esteban Moro[2,3], Manuel Cebrian[4,5,6], Nicholas A. Christakis[7,8,9], James H. Fowler[10,11]
1 Department of Computer Science, Escuela Politécnica Superior, Universidad Autónoma de Madrid, Madrid, Spain
2 Department of Mathematics & GISC, Universidad Carlos III de Madrid, Leganés, Spain
3 Instituto de Ingeniería del Conocimiento, Universidad Autónoma de Madrid, Madrid, Spain
4 Computer Science & Engineering Department, University of California, San Diego, CA, USA
5 Media Laboratory, Massachusetts Institute of Technology, Cambridge, MA, USA
6 National Information and Communications Technology Australia, Melbourne, Victoria, Australia
7 Department of Sociology, Yale University, New Haven, CT, USA
8 Department of Ecology and Evolutionary Biology, Yale University, New Haven, CT, USA
9 Department of Medicine, Yale School of Medicine, New Haven, CT, USA
10 Medical Genetics Division, School of Medicine, University of California, San Diego, CA, USA
11 Political Science Department, University of California, San Diego, CA, USA
∗ E-mail: Corresponding manuel.garciaherranz@uam.es

Contents

1 An Analytic Elaboration of the Friendship Paradox 2

2 The Twitter Data 4

3 Sensor Performance in a Simulated Infection Model 5

4 Sensor Performance in Real Data 7

5 Using the Sensor Method with a Small Set of Samples 8

6 Using the Sensor Method with Hashtag Networks 9

7 Reproduction Rates of Hashtags as a Factor Affecting Early Detection 11

8 Twitter, Sensors in Twitter, and Google Trends 12

9 Friends vs. Most Connected Nodes and Most Connected Friends as Sensors 14

10 Differences in Sensor and Control Characteristics That Also Affect Propagation 15

11 Figures 16

List of Figures

S1 The friendship paradoxes . . . 17
S2 Twitter data overview . . . 18
S3 Hashtags first appearance and popularity . . . 19
S4 Most used hashtags, users and Greatest Connected Component size . . . 20
S5 Variations of lead time of using friends as sensors with sample size for hashtags used by more than 0.01% of all users . . . 21
S6 Variations of lead time of using friends as sensors with sample size for hashtags used by more than 0.04% of all users . . . 22
S7 Variations of lead time of using friends as sensors with sample size for hashtags related to important events . . . 23
S8 Variations of lead time of using friends as sensors with sample size for different hashtags . 24
S9 Variations of lead time of using friends as sensors with sample size for hashtags for which the method works . . . 25
S10 Variations of lead time of using friends as sensors with sample size for hashtags for which the method does not work . . . 26
S11 Networks and cumulative distributions for hashtags with the biggest lead times . . . 27
S12 Networks and cumulative distributions for hashtags with big lead times . . . 28
S13 Networks and cumulative distributions for more hashtags with big lead times . . . 29
S14 Networks and cumulative distributions for hashtags with small lead times . . . 30
S15 Variations of sensor lead time with final number of hashtag users . . . 31
S16 Exposure rate of sensor and control populations . . . 32
S17 Variations of sensor lead time and divergence alarms with final number of hashtag users . 33
S18 Distribution of number of hashtag users for hashtags that trigger a divergence alarm . . . 34
S19 Distribution of number of users for hashtags triggering a divergence alarm vs. not triggering an alarm . . . 35
S20 Twitter hashtags and Using Friends as Sensors vs. Google searches . . . 36
S20 Continued. Twitter hashtags and Using Friends as Sensors vs. Google searches . . . 37
S20 Continued. Twitter hashtags and Using Friends as Sensors vs. Google searches . . . 38
S21 Lead time of using friends as sensors vs. using sensors by degree . . . 39
S21 Continued. Lead time of using friends as sensors vs. using sensors by degree . . . 40
S21 Continued. Lead time of using friends as sensors vs. using sensors by degree . . . 41
S21 Continued. Lead time of using friends as sensors vs. using sensors by degree . . . 42
S21 Continued. Lead time of using friends as sensors vs. using sensors by degree . . . 43
S21 Continued. Lead time of using friends as sensors vs. using sensors by degree . . . 44
S22 Degree and betweenness differences between controls and sensors . . . 45

1 An Analytic Elaboration of the Friendship Paradox

The "Friendship Paradox" was first elaborated by Feld (1991) who proved that, for any arbitrary social network (with variance in the degree distribution), the average number of friends of friends is greater than the average number of friends ("Your Friends Have More Friends Than You Do").

Mathematically, we can define μ as the average number of connections per vertex in a network with V vertices and E edges:

$$\mu = \frac{\sum_{v \in V} k_v}{|V|} = \frac{2|E|}{|V|} \tag{1}$$

Where each vertex $v \in V$ has k_v connections.

Using the same notation, we can derive the number of two-degree connections ρ (the friends of friends). Since the degree k_v of a vertex v is counted k_v times (if a person has 5 friends, each of them will count his friend 5-degree once, ending up being counted 5 times to the total number of friends of friends), there are $\sum_{v \in V} k_v^2$ two-degree connections, and we can divide this by the total number of direct connections $\sum_{v \in V} k_v$ to get the average number of two-degree connections ρ:

$$\rho = \frac{\sum_{v \in V} k_v^2}{\sum_{v \in V} k_v} \tag{2}$$

Using a property of the variance σ^2 in the distribution of k_v, note that:

$$\sigma^2 = E\left[(k-\mu)^2\right] = E\left[k^2\right] - \left(E\left[k\right]\right)^2 = \frac{\sum_{v \in V} k_v^2}{|V|} - \frac{\left(\sum_{v \in V} k_v\right)^2}{|V|^2} \tag{3}$$

From this, it is easy to show that:

$$\rho = \frac{\sum_{v \in V} k_v^2}{\sum_{v \in V} k_v} = \mu + \frac{\sigma^2}{\mu} \tag{4}$$

Therefore ρ is greater than μ (σ^2/μ times greater, to be precise) and this difference thus increases with the variance σ^2 of the degree distribution in the network.

Equation 2 implies that the expected distribution of two-degree connections $Q(k)$ will be a function of the degree distribution $P(k)$. Since each vertex of degree k is connected to k other vertices, each vertexs degree is counted k times in the distribution, leading to the following distribution of two-degree connections:

$$Q(k) = \frac{kP(k)}{\mu} \tag{5}$$

Since we want to sample only a part of the network of monitoring, we need to know the degree distribution we can expect to find for a random sample of a given size. If we sample a portion of the network ($0 \leq \gamma \leq 1$), the degree distribution $P(k)$ will remain the same since every person has the same probability γ of being chosen. However, the expected distribution of the two-degree connections for the "friends" sample will differ since a person with more friends has more chances of having a friend being chosen in the random sample and therefore appearing in the friends sample.

The probability of not choosing a person with k friends is equal to the probability of not choosing any of their k friends $(1-\gamma)^k$ and thus their probability of appearing in the friends sample is $1-(1-\gamma)^k$. Drawing on Equation 5, we can use these probabilities to derive the degree distribution of a random sample and a corresponding friends sample for a portion γ of the population:

$$\tilde{Q}(k) = \frac{k\left[1-(1-\gamma)^k\right]P(k)}{A_\gamma} \tag{6}$$

Where A_γ is a normalization factor.

For the complete network ($\gamma = 1$), Equation 6 simplifies to Equation 5, and both of these demonstrate the friendship paradox, $Q(k) > P(k)$ for large k. This effect is amplified for small γ since it maximizes the

multiplying effect of counting k times each vertex with degree k (see Supplementary Figure S1). Thus, a sensor group composed of the friends of a randomly-sampled control group will tend to have a larger number of high-degree vertices than the control group itself.

One issue with constructing a sensor group is that people with many friends may be friends of several persons of the randomly-sampled control group and therefore may be counted several times as members of the sensor group. In fact, a person with k friends will be counted k times when $\gamma = 1$. However, we remove such duplicates when constructing the sensor sample, so that each person is counted only once. This means that the degree distribution of the sensor group will not be multiplied by k:

$$\tilde{Q}(k) = \frac{\left[1 - (1-\gamma)^k\right] P(k)}{A_\gamma} \tag{7}$$

Notice that this sensor group with duplicates removed also exhibits higher *centrality* than the network as a whole since $\left(1 - (1-\gamma)^k\right)$ increases with k. However, like the friends degree distribution described in equation 6, the difference between the sensor group $\tilde{Q}(k)$ and the control group $P(k)$ in equation 7 disappears as γ increases (see Supplementary Figure S1). Thus, the centrality advantage of the sensor group declines as the size of the sample increases.

2 The Twitter Data

We used the data gathered by Kwak et al. [1], collected between the 1st of June 2009 and the end of December 2009, that represents a nearly complete graph collected by snowball-sampling the online social networking site, Twitter. Most (active) SNS users are connected to the giant connected component to which Paris Hilton belongs (a user with over one million followers who was used for the snowball sample). Additionally all users mentioning trending topics from June to August were also crawled. Summarizing, the sample includes:

1. those users connected to the giant connected component (no matter whether following or followed)
2. those who mentioned trending topics.

The data includes:

- 476,553,560 tweets
- 66,935,466 tweets using a hashtag (a key term prefixed by "#")
- 4,093,624 different hashtags
- 40,171,624 registered users
- 1,620,896 registered users using at least once a hashtag
- 1,468,365,182 follow relationships
- 531,703,974 reciprocal follow relationships (i.e. 265,851,987 bidirectional links)

The popularity of different hashtags, measured as the number of different people using each of them at some point during this period, approximately follows a power law. In addition, Supplementary Figure S2 shows a global increase in the number of tweets per day with a peak in summer as well as a weekly pattern in the number of hashtags used per day.

In Supplementary Figure S3, we show the popularity and start date for all hashtags appearing for the first time after June 25th, 2009. Since the start date for data collection is June 1st, 2009, and we did not observe these hashtags in the first 25 days, we assume that we have recorded their "birth" in the Twittersphere and therefore their possible initial outbreak.

We focus our analysis on the most used hashtags to analyze their virality in order to identify big local propagation communities. Supplementary Figure S4 shows the relative size of the greatest components for each network made up of all users using a particular hashtag, and compares it to the total number of users who used the hashtag.

3 Sensor Performance in a Simulated Infection Model

To test the theoretical behavior of the sensor mechanism in an environment with a known spreading process, we created a simple program in R to generate a synthetic Barabasi-Albert random undirected network with tail exponent $\beta > 3$ over which we simulated 10 cascades (each beginning at a different vertex of the network) and an infection-recovery process with infection probability $\lambda = 0.1$ and recovery probability $\gamma = 0.01$:

```
library(igraph)

# generate the network
g <- barabasi.game(50000,power=1,m=5,directed=F)
mm <- data.frame(get.edgelist(g))+1  #network edges
colnames(mm) <- c("id1","id2")

tend = 10000   # we simulate the model for 10000 time units

ntot <- max(c(mm$id1,mm$id2))

time <- rep(tend,ntot)    #infection times
infected <- rep(0,ntot)   #infected nodes (0=S, 1=I, 2=R)

ncascades <- 10      #number of cascades
lambda <- 0.1        #infection probability
gamma <- 0.01        #recovery probability

for(k in 1:ncascades){   # simulate 10 casacades
  i0 <- sample(1:ntot,1)  # select a random seed for the cascade
  time[i0] <- 0            # ...that gets initialy infected
  infected[i0] <- 1

  ni <- c(1)   # 1 node infected (just the seed)
  nr <- c(0)   # no nodes recovered yet

  for(i in 1:tend){  # begin the cascade
      #infecteds get recovered with probability gamma
      infected[infected==1 & (runif(ntot) < gamma) ] = 2

      ii <- which(infected==1) # get the list of still infected
      #neighbors of the infected nodes
```

```
      nb <- unique(c(mm[is.element(mm$id1,ii),2],mm[is.element(mm$id2,ii),1]))

      # Sucsceptible neighbors get infected with prob. lambda
      nb <- nb[runif(length(nb)) < lambda]
      nb <- nb[infected[nb]==0]
      infected[nb] <- 1

      time[nb] <- I  # record time of infection

      ni <- c(ni,sum(infected==1)) # update infected list
      nr <- c(nr,sum(infected==2)) # update recovery list
      if(ni[i]==0) break  # stop if there are no more infected
  }
}
```

We then used data generated by these simulations to collect 350 random samples of 7 different sizes (0.124% of the network to 12.5% of the network), 50 of each, and a similarly sized sample of its neighbors to use as sensor:

```
# function to get the neighbors of a node
neighbors <- function(id){
unique(c(mm[mm$id1==id,2],mm[mm$id2==id,1]))
}

samples <- c(62,125,312,625,1250,2500,6250) # sample sizes

final <- c()
for (samp in 1:length(samples)){ # for each sample size...
   dtc <-c()  # initialize control times
   dts<-c()   # initialize sensor times

   for(i in 1:50){ # repeat 50 times

      # get a control sample
      control <- sample(1:ntot,samples[samp])

      # and a sensor sample of its neighbors \wo repetition
      sensor <- sample(unique(unlist(lapply(control, neighbors))),samples[samp])

      # get the times of infection of the infected nodes
      tcontrol <- time[control]
      tsensor <- time[sensor]
      tcontrol <- tcontrol[tcontrol!=tend]
      tsensor <- tsensor[tsensor!=tend]

      # get the mean time of infection of controls
      dtc <- c(dtc,mean(tcontrol))
      #and sensors
      dts <- c(dts,mean(tsensor))
```

```
    }

    finalmean <-mean(dts-dtc) # mean Delta t
    finalesem <-sd(dts-dtc)/sqrt(length(dts)) # SEM Delta t

    #Save the mean and SEM Delta t of this sample size
    final<-rbind(final,c(as.character(sample[samp]), as.numeric(finalmean), as.numeric(finalesem)))
}
finaldf <- data.frame(final)
colnames(finaldf)<-c("sample","mean","sem")
```

The results can be seen in Figure 2a of the main text, showing that as the sample size grows, the SEM decreases, and the mean Δt approaches 0, suggesting that middle sized samples are the best choice for detecting significant lead times.

4 Sensor Performance in Real Data

We analyzed random control samples of various sizes (50K, 100K, 250K, 500K, 1M, 1.5M, 2M and 5M users), and compared each one against 30 random sensor samples of the same size. In these sensor samples we ensured that no user appeared more than once within a single sensor sample.

In order to detect a viral process with the sensor mechanism, two factors are crucial. First, the sensor sample should be more central than the control one. Second, the viral outbreak is large enough to be detected. We first study viral outbreaks in retrospect (once the spreading process is finished), focusing our attention on those that affected the largest number of nodes in the network.

In Supplementary Figure S5, all hashtags used by more than 0.01% of each samples users (or about 0.25% of all hashtag users) have been plotted and compared to the theoretical infection model. The results show that the mean Δt^{α} and variance approach 0 as the sample size grows, as predicted by the model. Supplementary Figure S6 shows the same thing for hashtags used by more than 0.04% of the sample (or about 1% of hashtag users). Notice that in both cases, small sample sizes are not large enough for lead times (Δt^{α}) to be reliably less than zero (due to sampling variation), while in large samples the control and sensor groups tend to overlap, causing the mean Δt^{α} to approach 0 (see Figure 2a,b in the main text).

Many of the important events of 2009 appearing on Twitter show a lead time for the sensor group. They also show a relationship with the control group by sample size similar to that of the infection model. For example, Supplementary Figure S7 shows hashtags for:

- *#h1n1*: Current level of the H1N1 influenza pandemic alert raised from phase 5 to 6, June 11
- *#indonesiaunite*: Suicide bombers hit two hotels in the center of Jakarta, July 17
- *#cop15*: UN Climate Change Conference 2009, December 7
- *#iran*: Protests following 2009 Iranian presidential election, June 13
- *#michaeljackson*: Michael Jacksons death, June 25.
- *#forasarney*: Social movement demanding the departure of Senator Jose Sarney of their duties in the National Congress of Brazil, June 17.

Supplementary Figure S8 shows that viral hashtags that grow in usage and spread from person to person over time, like *#mobsterworld*, and also those used in response to an exogenous event, like *#indonesiaunite*, show negative Δt^{α}, suggesting that the sensor method elaborated here may be a reliable

way to predict their widespread usage. In contrast, a few hashtags, like *#health*, yield a positive Δt^{α} (when the sensor group lags the control group). There is variation in the amount of lead time provided by the sensor group. Hashtags like *#beatcancer*, a one day campaign granting donations for each tweet using it, spread extremely quickly through the Twitter network. As a result, they display an almost flat curve around zero and no variance, suggesting that, in these cases, the sensor mechanism is not sensitive enough to predict an epidemic in advance.

Additional examples of viral and nonviral hashtags can be found in Supplementary Figures S9 and S10. We also show the network of users using a given hashtag and the cumulative distribution for the sensor and control groups for several example hashtags in Supplementary Figures S11-S14. Notice that, for many of these, the cumulative distribution of usage in the sensor group is shifted to the left, a sign that the center of the network is being infected before the network as a whole, on average, as expected.

5 Using the Sensor Method with a Small Set of Samples

Here, we study the use of multiple sensor and control samples for detecting contagious outbreaks. We choose 5 random samples of 50,000 Twitter users (to be used as control) with their complete set of followees (from which to obtain the sensor samples), calculating their in and out degree and the times at which they mention any hashtag in a tweet. Obtaining a complete list of followers from a random sample over the whole Twittersphere is a costly process, especially for large samples. Therefore we reduced our statistical analysis to 5 samples and followed the same methodology we later repeated with 1,000 samples when we did not need to tackle Twitter as a whole.

We trim the control samples to those users that have mentioned a hashtag at least once. Since only 4% of Twitter users have ever used a hashtag, our control samples are trimmed to about 2,000 users and the followees samples to about 150,000 users.

For each control sample, we randomly select a similarly-sized sensor sample (without duplicates) from its followees. We then remove all hashtags that have appeared before June 20th 2009 (20 days after our first records) reducing our analysis to those hashtags that were very likely "born" during our sample period. For each of the hashtags, we calculate the mean first time of use in the control and sensor samples. We then calculate the mean control time minus the mean sensor time for those hashtags used by at least 10 individuals in 1 or more of 5 samples.

Notice that the total population of these hashtags can be described with the following probability distribution:

$$P(\alpha, n_s, s) = \sum_{i=s}^{n_s} \left(\binom{n_s}{i} P(\alpha) i \left(1 - P\left(\alpha\right)\right)^{n_s - i} \right) \tag{8}$$

Where n_s is the total number of samples (i.e., 5) and s is the minimum number of samples (i.e., 1) in which we require the hashtag to appear in at least x_n users (i.e. 10). Therefore it is the probability of appearing in s samples and not appearing in the other $n_s - s$ samples multiplied by the number of possible combinations of n_s samples taken in groups of s, plus the same probabilities for every i sample where $s > i \geq n_s$.

Now, the probability of finding x_s or more users of a hashtag α in a random sample of S users drawn from a total population of N users is defined through the hypergeometric function, which indicates the probability of finding exactly x_s individuals of the X_α users of hashtag α in a random sample of S users drawn from a population of N individuals:

$$P(n_s; N, X_\alpha, S) = \frac{\binom{X_\alpha}{n_s}\binom{N - X_\alpha}{S - n_s}}{\binom{N}{S}} \tag{9}$$

The probability of finding x_s or more individuals is simply the cumulative distribution of Equation 9:

$$D(n_s; N, X_\alpha, S) = \sum_{k=n_s}^{X_\alpha} P(k; N, X_\alpha, S) \tag{10}$$

while the cumulative distribution for not finding $x_s - 1$ or less individuals is:

$$P(\alpha) \equiv D(n_s; N, X_\alpha, S) = 1 - \sum_{k=0}^{n_s-1} P(k; N, X_\alpha, S) \tag{11}$$

Where $P(\alpha)$ is the probability that hashtag α has at least x_s users in a random sample of S users drawn from a population of N individuals.

Complementing equation 8, equation 11 allows us to estimate the number of total hashtag users for hashtags with 10 or more users appearing in 1 or more of the 5 samples, 2 or more, and so on. The distribution of the total number of users of hashtags appearing in each of these cases and the distribution of their mean Δt can be seen in Figure 2c of the main text and Supplementary Figure S15.

Forcing a hashtag to have representatives in all 5 samples restricts our analysis to the most widespread hashtags (32 in our case), while lowering this restriction to hashtags that have enough representatives in at least one of the samples opens up the analysis to more hashtags (134 in our case) even though they do not necessarily have a global spread as wide as the former. In every analysis (i.e., hashtags having enough representatives in 1 or more of the 5 samples, 2 or more, 3 or more, 4 or more and in all of them), more than 70% of the hashtags show a negative Δt^α (across all analyses, mean 80.6% SD 7.3%).

Nevertheless, the results show that raising the threshold number of samples required for detection of the global spread of a hashtag reduces noise and yields a $\Delta t < 0$ for nearly all hashtags, providing advance warning of their outbreak. In other words, the wider audience a hashtag has reached, the greater probability that the sensor mechanism will detect it in advance.

6 Using the Sensor Method with Hashtag Networks

Given the comparatively low number of hashtag uses on Twitter (about 14% of the tweets and only 4% of the users use hashtags) and the high diversity of hashtags (4 million different hashtags for 66 million tweets displaying a hashtag), the probability of finding enough users of a hashtag for statistical analysis in a small random sample of Twitter is very small. We note, however, that a focus on hashtags per se does not limit the generality of the methods and findings we describe.

Therefore, to analyze particular hashtags, we focused on the complete network of their users, composed only of those people who have used the hashtag in at least one tweet, and their follow relations. This allows us to individually analyze the dynamics of well-known hashtags as well as to try to identify smaller networks through which a viral process has spread.

We selected all hashtags that were used more than 20,000 times on Twitter, identified their users and their follow relations to construct, for each of them, its hashtag network. For each of these hashtag networks, we selected a random control sample of 5% its size and a similarly-sized sensor sample of their followees to calculate Δt^α. We then repeated this process 1,000 times to generate a statistical distribution of these observed lead times (Figure 2b of the main text). The sensor group led the control group ($\Delta t^\alpha < 0$) 79.9% (SE 1.2%) of the time (52.9%, SE 1.1% for $\lceil \Delta t^\alpha_{p-value=0.05} \rceil < 0$). However, note that there was considerable variation in lead times, from 20 days to a few hours or no advance warning.

Moreover, one could use the difference in the sensor and control group to determine whether or not a viral process is under way. For example, estimating the models each day using all available information

up to that day, for a control and a sensor sample of *#openwebawards* users, we find a significant lead time ($p < 0.05$) on day 13, a full 15 days before the estimated peak in daily incidence, and also 15 full days before the control sample reaches the same incidence as the sensor group has on day 13 (See Figure 3c in the main text and right figures of Supplementary Figures S11-S14).

While these results are promising, a Δt^{α} does not necessarily indicate a viral process. Consider a non-viral hashtag with fixed probability of use λ. A user tweeting at a rate of 10 tweets per hour will use a hashtag with probability 10λ in the first hour, 10 times bigger than that of a user tweeting at a rate of 1 tweet per hour. Due to the correlation between degree and tweet frequency (see Figure 4a in the main text) a $\Delta t^{\alpha} < 0$ could be observed for a hashtag not because of its virality but because of the potentially higher tweeting rates of users in the sensor group.

To see how the sensor method works for hashtags that are *not* spreading virally, we generated a null distribution in which we randomly shuffled the time of each hashtag use within the fully observed data, and then measured the resulting difference in the sensor and control group samples, $\Delta_R t^{\alpha}$. This method preserves any effect that correlation between degree and activity level might have on lead times. Again, we repeated the procedure 1,000 times to generate a statistical distribution. The results show that the observed distribution of lead times fall outside the null distribution for 65.4% (SE 1.2%) of the hashtags (25%, SE 0.95% for $\lceil \Delta t^{\alpha}_{p-value=0.05} \rceil < \lfloor \Delta_R t^{\alpha}_{p-value=0.05} \rfloor$), suggesting they did, in fact, spread virally.

Therefore, in the shuffling process sensors actually have a greater chance of getting smaller times of infection than controls because they have more tweets to be assigned a new timestamp. By shuffling the timestamps of every tweet we are measuring the lead time sensors would get not because of their centrality in a viral process but because of their higher tweeting rates. The difference, therefore, between this lead time and the observed one corresponds to the viral component of the process.

Notice that this method gives us greater confidence that the sensor method detects viral processes, but it also suggests that the non-viral component of Δt could help the sensor mechanism work better than expected, since sensors not only get reached sooner by an epidemic (due to their centrality) but also react earlier (due to their higher tweeting rates).

Another plausible explanation to why high degree users manifest hashtags before the hastags trend might be that they are novel information sources, meaning they use hashtags before they become popular and that, because of this, they gain attention and followers. Or, from another perhaps more machiavellian perspective, that they use their privileged position in the network to promote their own hashtags. In other words, that central actors *select* novel topics rather than being agents of contagion.

In order to evaluate this possibility, we calculated the exposure rates of sensors and controls as $\epsilon = N_{exp}/N$ for all hashtags in Figure 3a of the main text, where N_{exp} is the number of users who used the hashtag after being exposed to it, meaning that at least one followed user used the hashtag before him, and N is the number of users in the sample. We repeated this process 1,000 times per hashtag choosing each time a new random control sample and a similarly sized sensor sample of its followees to gain statistical significance.

The results can be seen in Supplementary Figure S16 and show that the exposure rate is significantly higher in the sensor group, ruling out the selection idea in favor of the contagion hypothesis.

These results support the idea that sensors are good transmitters in Twitter (they are aware of whats happening in Twitter and transmit it very soon) while controls seem to introduce more information in Twitter from other sources (or create it), rather than transmitting what they are exposed to in Twitter, though this information has been around in Twitter for a long time most of the time (hence the negative Δt).

7 Reproduction Rates of Hashtags as a Factor Affecting Early Detection

Everything that can be mimicked or passed along by exposure can be thought of as viral, and therefore all hashtags that can be seen and copied can be considered viral to some degree. Nevertheless, accurately establishing to what degree a particular hashtag is more or less viral than another one is a complicated task.

While we may think of a hashtag as "viral" if it is used by many people, it is worth noting that this definition of virality leaves aside very important factors such as target population size. For example, we might categorize a mild flu affecting all species in a zoo with a contagion probability of 0.01 to be more viral than a killing virus exterminating all monkeys in a matter of hours with a contagion probability of 0.9, just because the latter only affects monkeys and therefore the final number of affected animals will be smaller. On the other hand, given that viral processes are occasionally very powerful, it is possible that many high-incidence outcomes can be explained by contagion.

The fact that widely spread hashtags tend to have a strong viral component can be seen indirectly with the sensor mechanism. Consider a simple equation traditionally used to measure the transmission potential of a disease, $R_0 = \lambda k$, where R_0 is the basic reproductive rate (or number of secondary cases) of an infectious process, λ is the infection probability, and k the average number of contacts (i.e., degree). Under this formulation, R_0 is increasing in both the rate of infection (bigger λ) and the connectivity of the network (bigger k), and, as R_0 grows, so does the chance that a disease will create an epidemic. For highly viral processes with big R_0, we should expect central users to show a positive lead infection time but not as big as that of less viral processes since the epidemic travels faster and therefore the difference between times of infection of central and random samples should be smaller.

Supplementary Figure S17 shows that those hashtags that end up being used by the most users are, in fact, more likely to exhibit smaller lead times (i.e negative Δt) suggesting that they spread via faster, more contagious processes (bigger λ). Therefore, we see that highly diffused hashtags behave as processes with high reproduction rates. It is nevertheless important to recall that this does not work the other way around, meaning that while highly viral processes display mild lead infection times of central samples, processes showing this behavior are not necessarily highly viral.

In Supplementary Figure S17, we also introduce a concept called a *divergence alarm.* Instead of merely relying on Δt (i.e. mean time of the central sample minus mean time of the random sample) as a lead time measure after the fact, we can monitor real time differences between the sensor group and the control group as an epidemic unfolds. If the rate of infection in the sensor group is growing faster than the control group, it suggests that a hashtag is spreading from person to person (compared to a broadcast mechanism where individuals in the sensor and control group should be equally affected) and therefore it may eventually be one of those hashtags with the highest number of total uses. We trigger an alarm if, at some point, the percentage of infected nodes in the sensor sample is X% higher than that of the random sample.

To test this alarm, we studied the dynamic spread of 2,000 moderately used hashtags ranked from 4,000 to 6,000 in their overall total usage. We did this instead of analyzing the most used hashtags because while having a significant and similar amount of uses (between 1,800 and 1,100) there is a wide variety of final number of users (from 3 to 1,047) which cannot be found in the top used hashtags, thus providing a perfect benchmark for testing the sensor mechanism and how it is able to distinguish hastags that end up being used by a significant amount of people from those that does not. For each hashtag we chose a random control population of 10% the size of the final total usage. We specify that we require a control population of at least 10 users and a sensor one of at least 5 to proceed with the analysis, otherwise the hashtag is discarded and no early warning signal is triggered. For those hashtags with enough users in the control and sensor samples (1,111 out of 2,000) we analyze at which point the sensor mechanism triggers a divergence alarm for different thresholds.

As we can see in Supplementary Figure S17, for an alarm threshold of 1%, an alarm is triggered at some point for most of these 1,111 hashtags, mainly those showing a positive mean lead time in the sensor group. In contrast, the 133 cases where no alarm is triggered are equally distributed, with some showing positive and others showing negative lead times.

The distribution of "no-alarms" depends on the threshold of choice for the divergence alarm as can be seen in Supplementary Figure S18. A threshold of 0.25% should capture more and faster processes in which central users act as hubs that are quickly followed by peripheral ones, creating a divergence in incidence curves. However, the trade off is that this more sensitive alarm triggers for more hashtags that have smaller lead times (as should be expected from high reproductive rates: R_0). In fact, for a threshold of 0.25% only 11 out of the 1,111 hashtags trigger no alarm. By increasing the threshold we miss out on fast processes while still triggering those viral processes that are slow enough to generate a significant core of cases in the central population before spreading to the rest.

Supplementary Figure S18 also shows how, as we increase the alarm threshold, the density of "no-alarms" increases in the most-used-hashtags region of the plot, suggesting that the density of hashtags with high reproductive rates (R_0) grows with the number of total uses of the hashtags (as shown in Supplementary Figure S17 as well).

Overall, we are interested in seeing if the sensor mechanism can identify which hashtags will end up being used by a significant number of users. Despite the slight variation in results due to the different thresholds used for triggering divergence alarms and the relaxed condition of finding at least 10 controls and 5 sensors using the hashtag to trigger the analysis, we observe in Figure 4b of the main text that the distribution of the final number of users for those hashtags in which the sensor mechanism has triggered an alarm is extremely shifted to the right. That is, hashtags that trigger alarms tend to end up being used by more users than hashtags that do not trigger alarms. Supplementary Figure S19 further shows how these distributions change for various choices of divergence threshold.

8 Twitter, Sensors in Twitter, and Google Trends

Are Twitter trends seen earlier than through any other media? The sensor mechanism has so far pointed at the possibility of early detection within Twitter but it is natural to question whether or not this anticipation is also valid for the real-time unfolding of the event in other contexts. This question could be asked even more generally, of course, by considering whether Twitter itself is a leading or lagging indicator of what is happening in the world – a question with both methodological and phenomenological significance. Ideally, contagions detected with the sensor method deployed in Twitter would not only be detected early with respect to Twitter (which, as we have shown, they are), but would also be detected earlier with respect to events occurring outside Twitter.

In comparing hashtags with real-time events we must consider that:

1. Not all hashtags are clearly tied to external events (for example, consider *#indonesiaunite* versus *#onletteroffmovies*).

2. We need to be sure that we correctly identify the correspondence between the hashtag and the event as referred to in other media (*#jobs*, for example, is used in Twitter to post job offers while "jobs" in Google is queried to find open job positions or information about "Steve Jobs").

Using Google Trends as an outside-Twitter source of information, we have selected a random sample of important hashtags that also have a clear correspondence in Google Trends.

Among the more than 2.5 million hashtags, only a few of them have been used more than a couple of times, only 24,000 have been used more than 200 times, and only 3,696 of these more than 2,000 times. This means that most hashtags are almost non-existent and will not be detected by any media or by our

sensor mechanism. Therefore we have restricted our analysis to the top 3000 most used hashtags (their total usage ranges from 2,000 to 90,000 times). From this list we have selected 16 random hashtags that:

1. Correspond to a range of important events including politics, health, social revolutions, product releases, or celebrities. We specifically exclude events created by or for Twitter, despite their importance (such as *#FollowFriday*, *#mobsterworld*, *#wheniwaslittle*, or *#twitterafterdark*) since they will not appear in Google Trends.

2. Are specific enough to be matched with some particular event since, for generic topics such as *#politics*, *#music*, *#tech* or *#obama*, it is nearly imposible to narrow down their equivalent in Google Trends to some meaningful particular. E.g. *#politics* is used in tweets as varied as:
   - "Afghan Violence Hits All-Time High"
   - "A rising tide lifts all boats. Proverb"
   - "President Obama has developed a general disregard for t..."
   - "Harry Reid Euthanizes Pet Project"
   - "Today's Obliviousness Award..."
   - "Chinese 'porn blocker' just a ruse"
   - "Wright says 'Jews' keeping him from Obama"
   - "Real Reasons Miss Calif. Got The Boot"
   - "See #TonyBlair 's maiden speech: http://"
   - "The right to keep and bear arms isn't limited to firearms"

3. The equivalent Google search shows a search peak in Google Trends in the second half of 2009; that is, there has been an outburst of public attention in the non-Twitter world within the period for which we have Twitter data.

We manually curated Google searches to compare to each hashtag since automatic translations often lead to noisy data. For example, according to http://tagdef.com, the hashtag *#gr88* "stands for green revolution and the Persian calendar year is 1388 hence gr88" and would ideally be matched with "green revolution". Nevertheless, this term might be confused with the agricultural "green revolution" which sees search peaks related to Norman Borlaugs death or Bill Gates call for a revolution in agriculture, so we chose "Iran green" instead, which, according to Google Trends, is related to news such as "In Iran green has become the color of protest".

Figure 4a, in the main text, shows nomalized Twitter trends in randomly-chosen control and sensor groups and compares them to normalized Google searches over the same period. Additionally, we show the points in time when the sensor and control distributions indicate a 2.5% and 5% divergence, both of which are even stricter criteria than those we tested above.

In Supplementary Figure S20, we observe that, in general, Twitter (green dashed line) behaves like Google searches as measured by Google Trends (blue line), usually with some anticipation in the peak, with the exception of *#iranelection*, which could be due to a slight difference in subject matter since, in Twitter, the hashtag is associated with Irans revolution and peaks around 21 June 2009 (concurrently with *#neda*, *#gr88* and *#iran*) while in Google Trends the search term "Iran elections" might be associated with news about elections in Iran, which took place on 12 June 2009.

Despite the usual slight anticipation of Twitters peak compared to the one in Google Trends, a divergence between the sensor (thin red dotted line) and control (blue dashed line) cumulative distributions can be observed several days to up to many weeks in advance of the peak (orange and red dotted lines for 2.5% and 5% divergences, respectively), meaning that the sensor mechanism serves as an early warning signal for *both* Google Trends and Twitters peak. The anticipation of the sensor mechanism is minimized in responses to unanticipated sudden events such as Michael Jacksons death, the Jakarta bombings, or Nedas killing (see Supplementary Figures S20-J, S20-K, and S20-O).

9 Friends vs. Most Connected Nodes and Most Connected Friends as Sensors

The friendship paradox serves as a proxy to get a central population without having to analyze the complete network. As the network size grows, analyzing the whole network becomes an intractable problem and therefore having a proxy for more central users might be the only real option available, but how good are these friend groups compared to central groups obtained through global analysis?

To answer this question, we checked the variation in lead times for sensor groups of different mean degree, comparing them to the lead time observed when using the friendship method. This analysis was conducted over the hashtag user networks described above (i.e., all users that have ever used a particular hashtag) of Figure 3a of the main text.

For each hashtag network in our list of most used hashtags, we selected a control sample of 5% the size of the network and a sensor sample to calculate Δt.

Then, after ordering the hashtag users by their degree (i.e., number of people that follow a user), we took samples of size n (where n is the number of users in the control sample) in the following manner: the first sample is composed of the n users with the highest degree, the second sample is the n users with the highest degree after removing the user with the highest degree, the third is the n users with the highest degree after removing the two highest degree users, and so on until the last sample which is the n users with lowest degree. Therefore, there are $N - n$ overlapping samples where N is the number of hashtag users. We will refer to these samples as k-groups (as they are groups with a decreasing degree k).

We then computed Δt for each k-group, that is, mean time of first use of the hashtag in the control group minus mean time of first use of the hashtag in each k-group. This process is repeated 20 times (with 20 different random groups as control) to evaluate statistical significance.

Supplementary Figure S21 shows how Δt varies (one plot per hashtag) with the mean degree of the k-group.

We ordered these plots from highest to lowest average Δt (as shown in Figure 3a of the main text), beginning with *#lightupnigeria* (the hashtag for which we observed the highest Δt) and ending with *#iamblessed* (the one with the worst response to the sensor mechanism). On each plot horizontal dashed lines and dotted lines mark the $\Delta t = 0$ and the sensor-sample mean Δt, respectively. The vertical lines mark the mean degree of the control and friend samples, respectively.

As expected, we observe that Δt gets smaller as the mean degree of the sensor group increases – the higher the mean degree of a group, the sooner it gets infected (i.e., uses the hashtag). Only *#beatcancer* shows an opposite signature in which high degree nodes are last to get infected, but some hashtags such as *#itsnotgonnawork* or *#iamblessed* show more irregular behavior.

Supplementary Figure S21 also shows that low degree groups are generally more inconsistent than high degree groups in terms of time of infection, leading to greater variation on the left side of the plots compared to the right.

Finally, while there are some cases in which choosing a high degree group would have improved the otherwise good results of choosing friends as sensors (e.g. *#nevertrust*, *#indonesiaunite*, *#openwebawards*, *#wheniwaslittle* and *#in2010*), most of the time the sensor mechanism gives a similar result (e.g. *#whentwitterwasdown*, *#pengakuan*, *#famousexcuses* or *#itmightbeover*), a better one (e.g. *#lightupnigeria*, *#fun140*, *#onletterofmovies* or *#wecantdate*) or even better than any of the k-groups available (like in the case of *#twitdraw* in which the sensor sample Δt of -10 days falls outside the plot).

Although users with higher degrees might improve the lead time observed in some cases, calculating the most connected nodes of a network is very expensive computationally, and calculating other types of centrality may be intractable. Using friends as sensors is, conversely a straightforward process that can be nevertheless improved or refined by choosing the most connected friends, therefore raising the mean degree of the sensor group while focusing the analysis on a much smaller set of users.

In particular, note that all the analysis here has been done using the *a-posteriori* known networks of hashtag users. However, these networks are not known *a priori* when crawling Twitter and therefore, to obtain a higher degree sample without using the friendship paradox, one would need to randomly choose a bigger sample from which to select the most connected nodes. And the size of this sample would need to be much larger than the sample needed to implement the method via the friendship paradox. To see why, consider these facts:

- A control sample of 50,000 random users follows about 700,000 others.
- Only 4% of Twitter users ever use a hashtag so, from our original control sample of 50,000 users, only about 2,000 users have ever used a hashtag.
- Thanks to the friendship paradox, the 700,000 followees of these users are more central, and since there is a positive correlation between degree and hashtag use, about 25% of them use a hashtag (i.e., about 180,000 users), rather than the 4% of the random sample.
- In order to get an equally sized sample of hashtag users through a random mechanism we would have to start with a sample of 5,000,000 users from Twitter (about 12% of the whole Twitter database in 2009), of which 4% (i.e. about 180,000) would have ever used a hashtag.
- Even when obtaining 180,000 hashtag users by sampling 5,000,000 random users in Twitter, we find that its 50,000 most connected users have a mean degree about 6 times smaller than the top 50,000 hashtag users of the followees sample.

Summarizing, to implement an alternative method that focuses on high degree users, we would need a sample more than 5 five times greater than when using the friends method to obtain an equally sized sample of hashtag users that is, nevertheless, 6 times less connected!

In addition, when we choose the sensor group from the friends of the control group, we are focusing the analysis in a connected (and therefore locally meaningful) part of the network. As a result we find more hashtags that are used both in the sensor and control samples and that are, consequently, prone to analysis. Finally, by using the friends-as-sensors method, we are able to probe many regions of the overall network, something that would be less likely if we focused on high degree, central users who, by definition, occupy a central part of the network.

10 Differences in Sensor and Control Characteristics That Also Affect Propagation

By construction, control and sensor groups differ in the local structure of their networks. This is reflected in properties such as degree, betweenness or k-coreness. For instance, in the *#openwebawards* user network, for 30 control/sensor samples of 10% its user base, sensor users have a mean degree of 24.6 (SEM 1.4), 6.6 times greater in average (SEM 0.6) than the 4.5 (SEM 0.6) mean degree of control users, and a mean betweenness centrality 6.2 times greater too (SEM 0.4), 17,615.9 (SEM 492.9) compared to the 3,119.7 (SEM 192.5) of the controls. For 340 random control and sensor samples of 34 different hashtag networks (from 4,645 to 108,073 users) in only 3 cases did the sensor group have a lower mean degree than the control group. Nevertheless, in all of them the sensor group had a higher mean betweenness than the control one (see Supplementary Figure S22).

However, this is not the only factor affecting how much a user may contribute to a contagious outbreak. They may also exhibit different patterns of communication that contribute to the spread of information from person to person to person.

From a random sample of 1,000,000 users, we identified a control group of 36,499 users using at least one hashtag and a sensor group of the same size composed of the control groups followees. After removing

users from the control group that are present in the sensor group, we end up with 16,332 users in the control group.

Figure 4c of the main text shows how the sensor and control groups differ by degree. The mean number of followers and followees are 25 and 28 in the control group, respectively, but they are 422 and 243 in the sensor group. These numbers reiterate the main result from Figure 1 of the main text, that sensor samples tend to be much more central than control samples. But there is also a large difference in communication behavior. The sensor group tweets more (152 tweets per user compared to the control groups 55) and also uses more hashtags per tweet (26 compared to the control groups 9). Both of these comparisons show that the sensor group is about three times more active than the control group, but Figure 4c shows that this is a trivial consequence of the relationship between number of tweets and number of connections. In both the control group and the sensor group, the better connected you are, the more messages you send.

Although the *quantity* of communication is the same for the sensor and control group once we control for differences in connectivity, we find a difference in the *quality* of these messages net of the difference in activity levels. Since hashtags are used to denote topics, we can study the extent to which users participate in many different topics or just a few.

Analyzing the number of different hashtags used per tweet and the number of unique hashtag uses, we find a different trend between the control and sensor samples (see Figure 4d in the main text). Sensors use each different hashtag 2.53 times on average (95 percent confidence interval: 2.23 2.83), slightly more than control users who use each hashtag on average 2.15 times (95 percent confidence interval: 2.03 2.28). Nevertheless, as we have shown, sensors tweet significantly more than controls and when controlling for the *frequency* of hashtag usage (hashtags per tweet), we find that users in the sensor group tend to use a significantly wider *variety* of hashtags than control users (i.e. they get involved in a larger number of topics), 96.85 different hashtags per tweet (95 percent CI: 93.51 to 100.20) compared to 38.66 different hashtags per tweet for the controls (95 percent CI: 37.47 to 39.85).

This fact could be of special importance when using the sensor mechanism to anticipate hashtag epidemics as sensors act not only as social hubs (by having more connections) but also as faster responders (by tweeting more) and as information hubs (by being involved in more topics).

References

1. Kwak H, Lee C, Park H, Moon S (2010) What is twitter, a social network or a news media? In: Proceedings of the 19th international conference on World wide web. ACM, pp. 591–600.

11 Figures

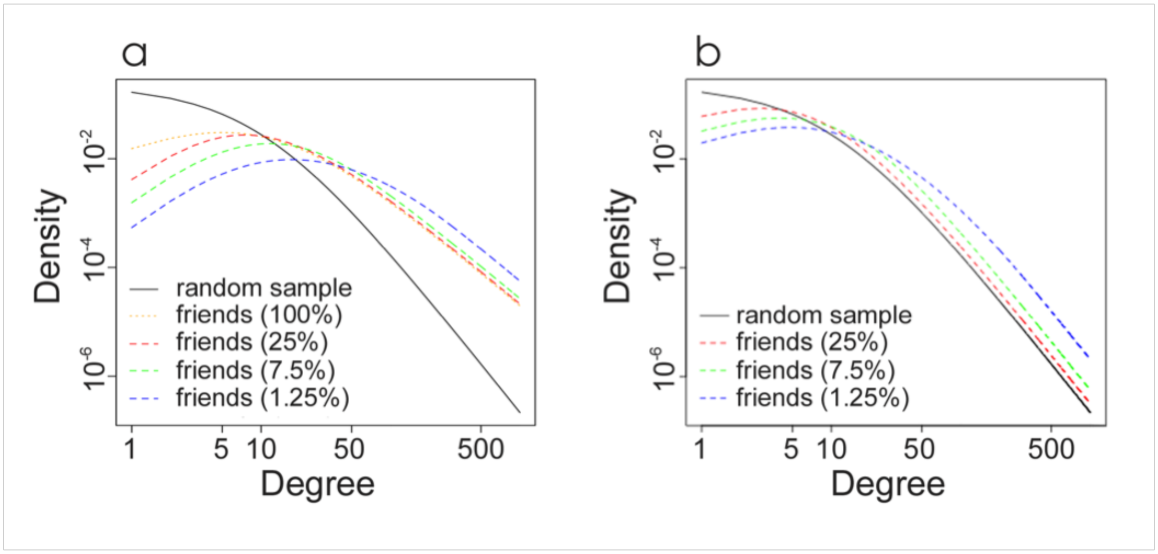

Figure S1. Larger samples of friends (as a percentage of the total number of users in he network) show a smaller difference in degree distribution from the overall network both for a) groups of friends with duplicates (e.g. a friend of two users is counted twice) and a) groups of friends without duplicates (a friend of several users is counted only once).

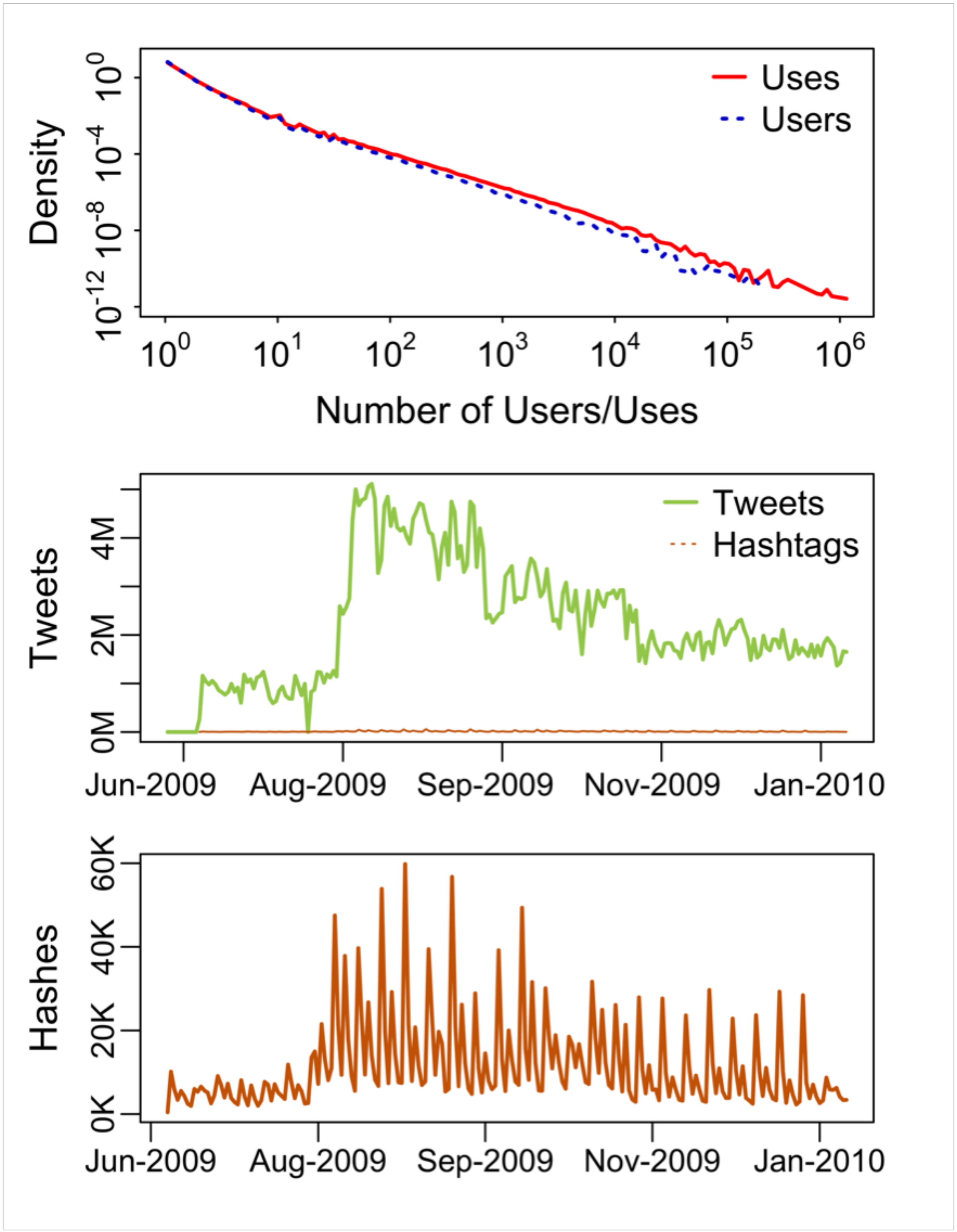

Figure S2. Popularity distribution of all hashtags appearing between June 2009 and January 2010 measured as the number of users of each hashtag. This distribution approximately follows a power law. The bottom plots show the total number of tweets and hashtag uses per day.

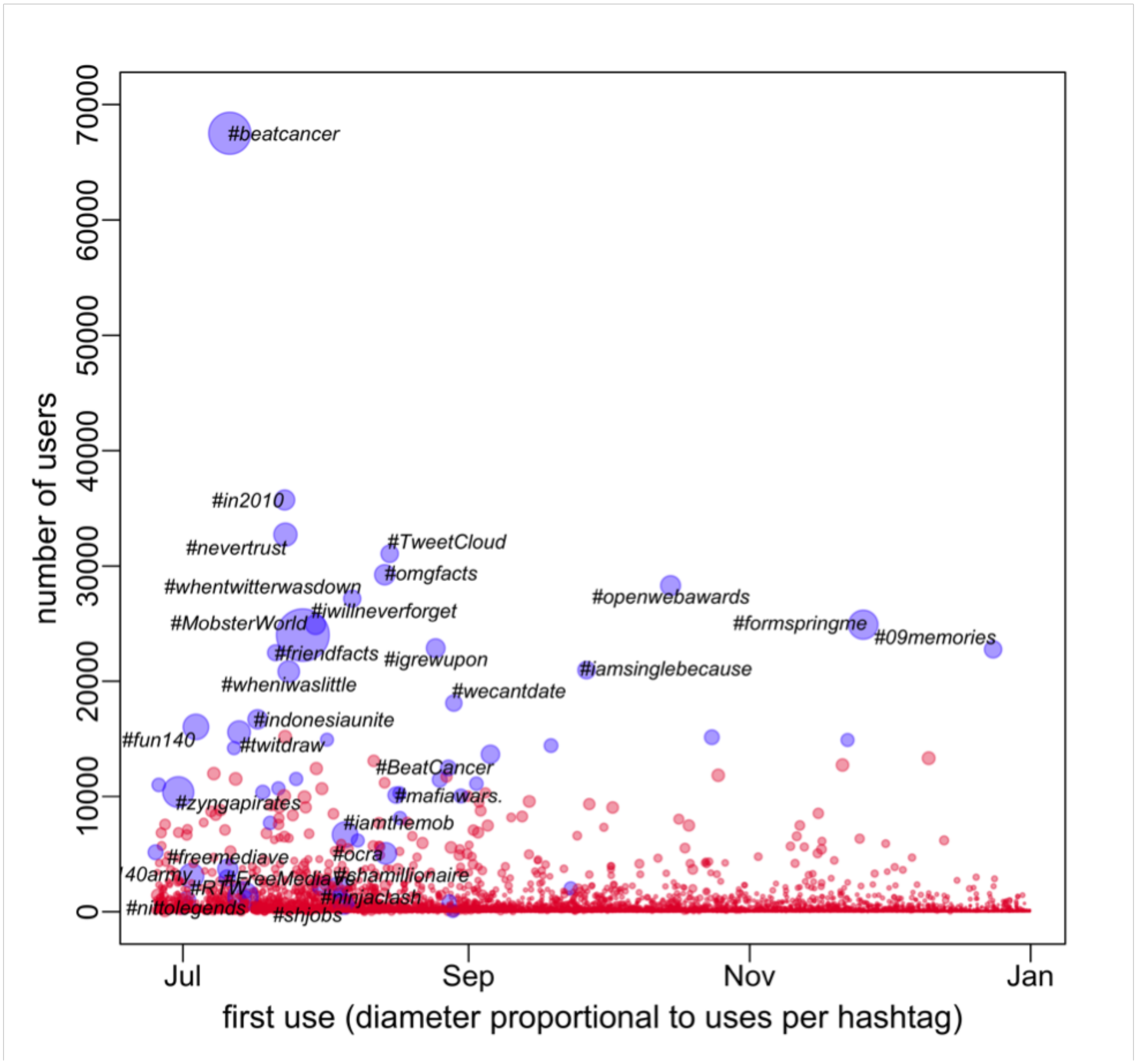

Figure S3. Appearance time and number of users of all hashtags appearing for the first time after June 25th. The diameter is proportional to the number of times the hashtag has been used. In blue are all hashtags used more than 20,000 times.

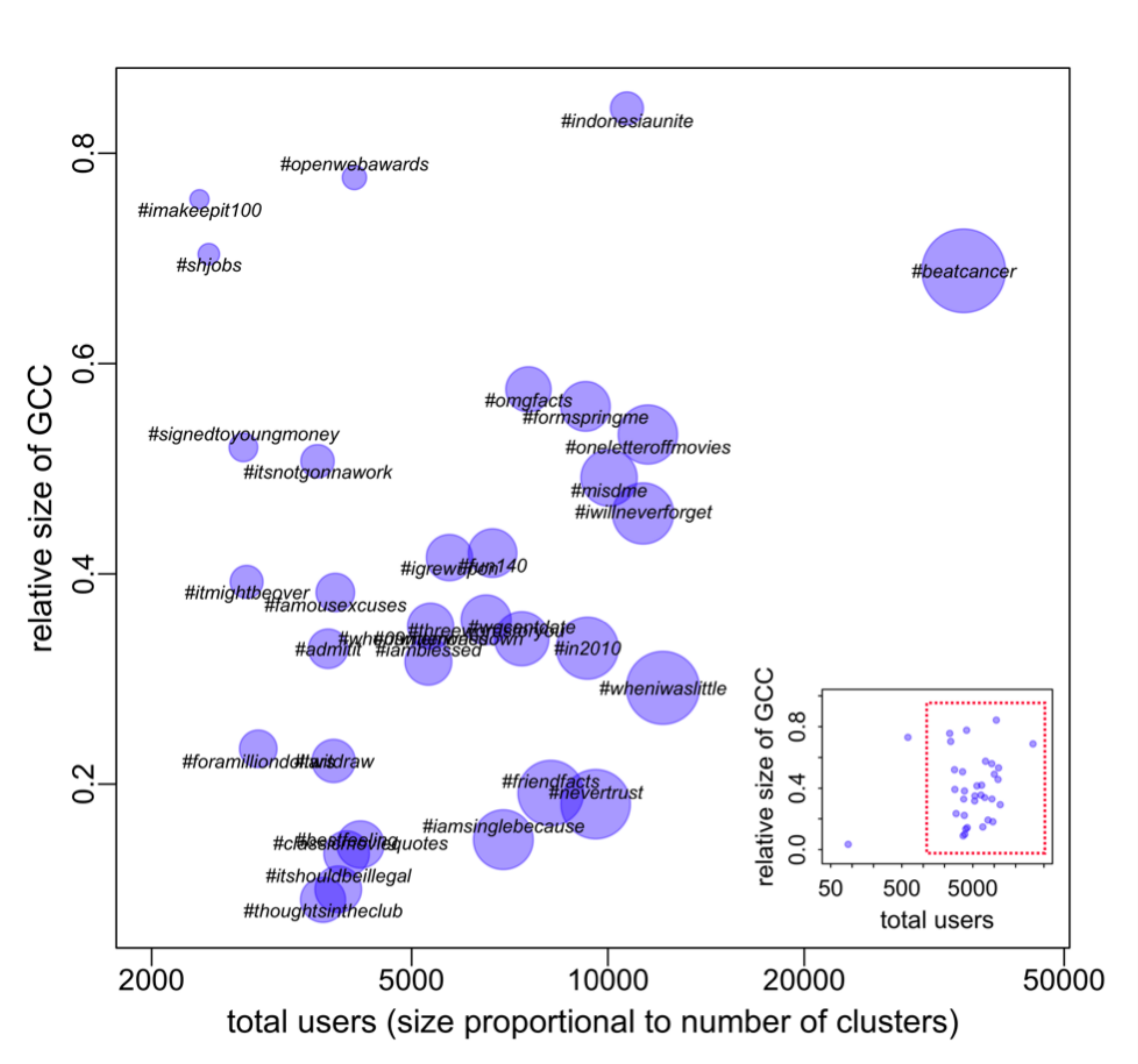

Figure S4. Number of nodes in each hashtag network (composed of all users of a particular hashtag and their follow relations to others using the hashtag) and relative size of its greatest component for all hashtags used more than 20,000 times (those in blue in Supplementary Figure S3). The size of the nodes is proportional to the number of components of the network. Most networks show a large greatest component and many small isolated components.

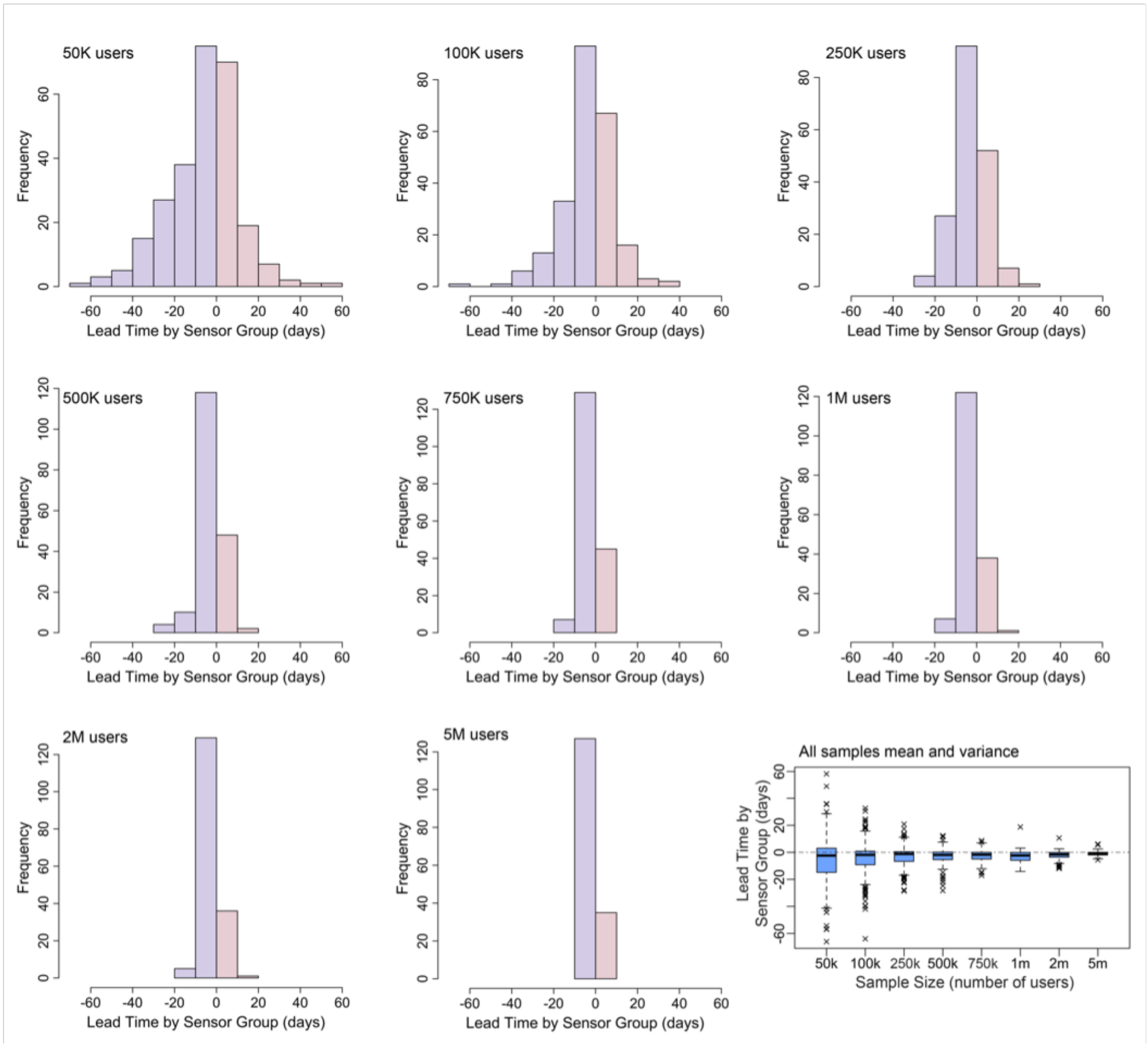

Figure S5. Sensor lead time (Δt^{α}) distribution for hashtags used by more than 0.01% of all users in various sample sizes.

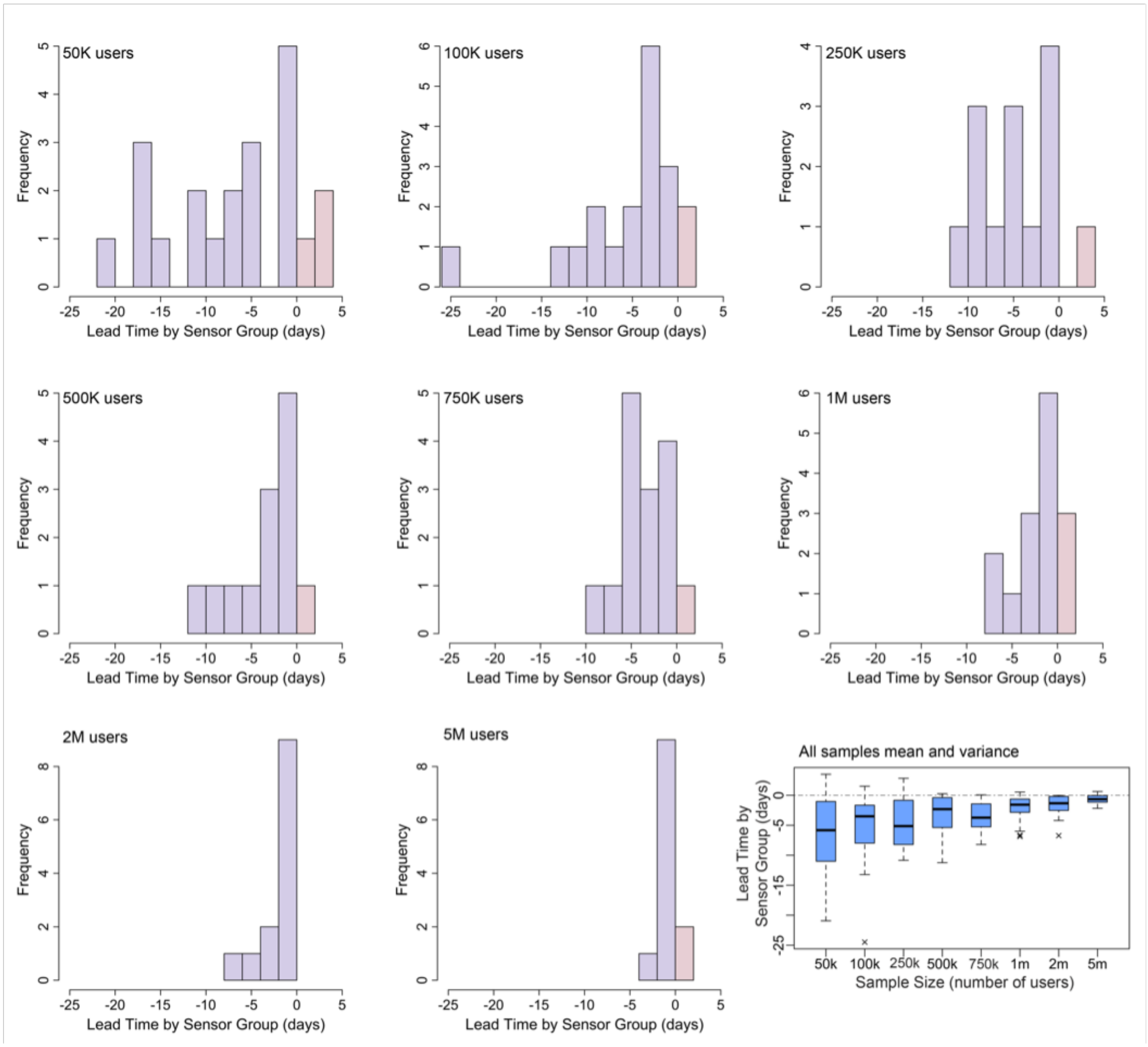

Figure S6. Sensor lead time (Δt^{α}) distribution for hashtags used by more than 0.04% of all users in various sample sizes.

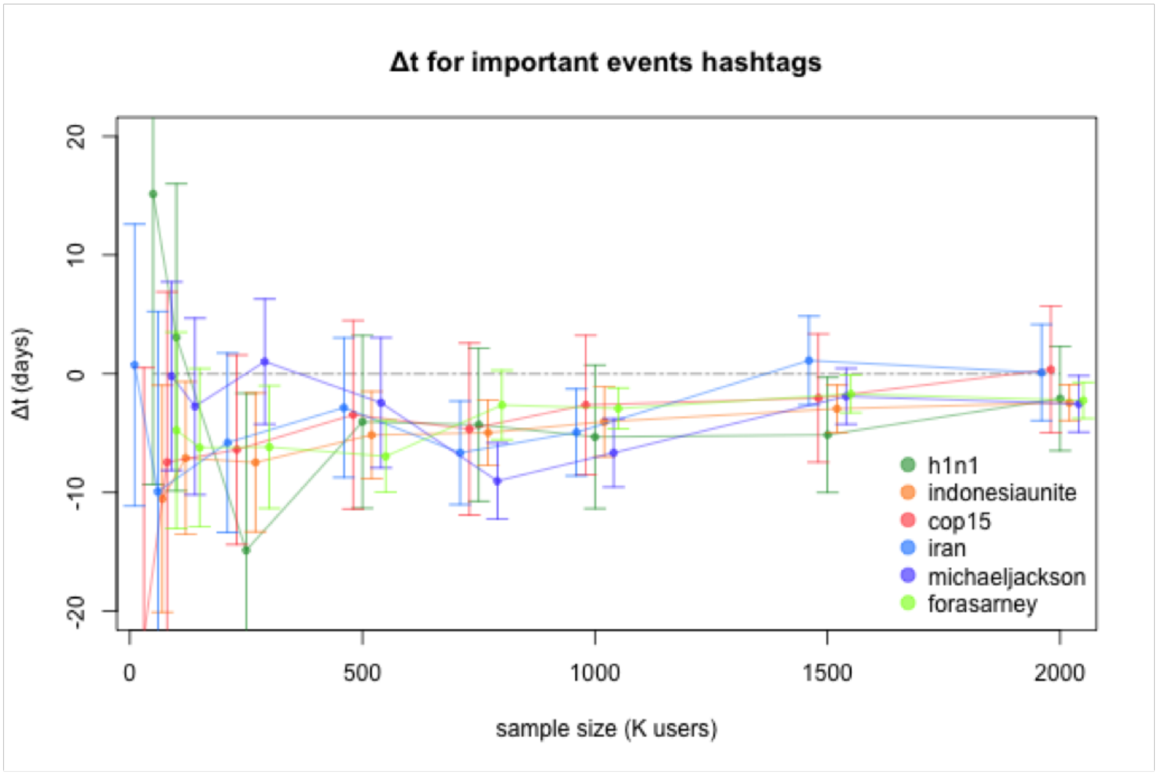

Figure S7. Lead times in the sensor group compared to the control group (Δt^{α}) in different sample sizes for hashtags related to important events in 2009.

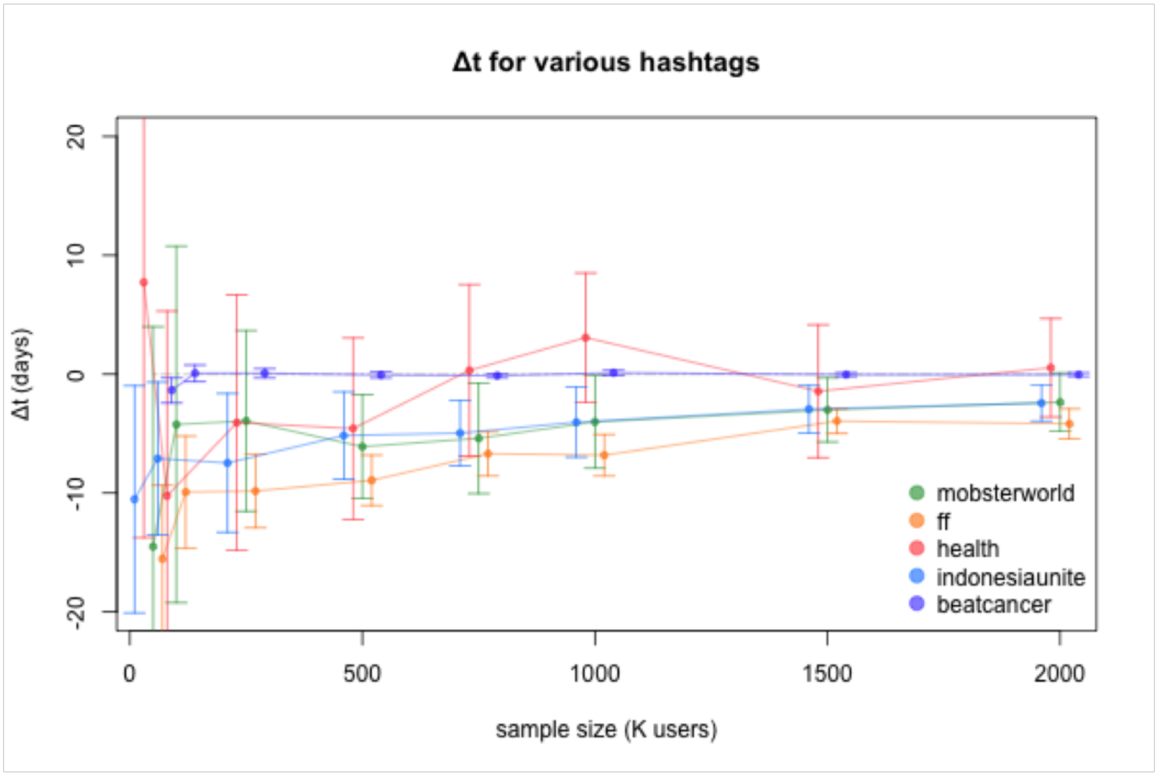

Figure S8. Lead times (Δt^{α}) for different control samples for *#mobsterworld*, an endogenous viral hashtag; *#ff*, which despite its non virality shows a $\Delta t^{\alpha} < 0$ similar to that of the simulated infection model on the synthetic network; *#health*, a nonviral hashtag, and *#indonesiaunite* and *#beatcancer*, two hashtags for which the sensor mechanism respectively works and does not work.

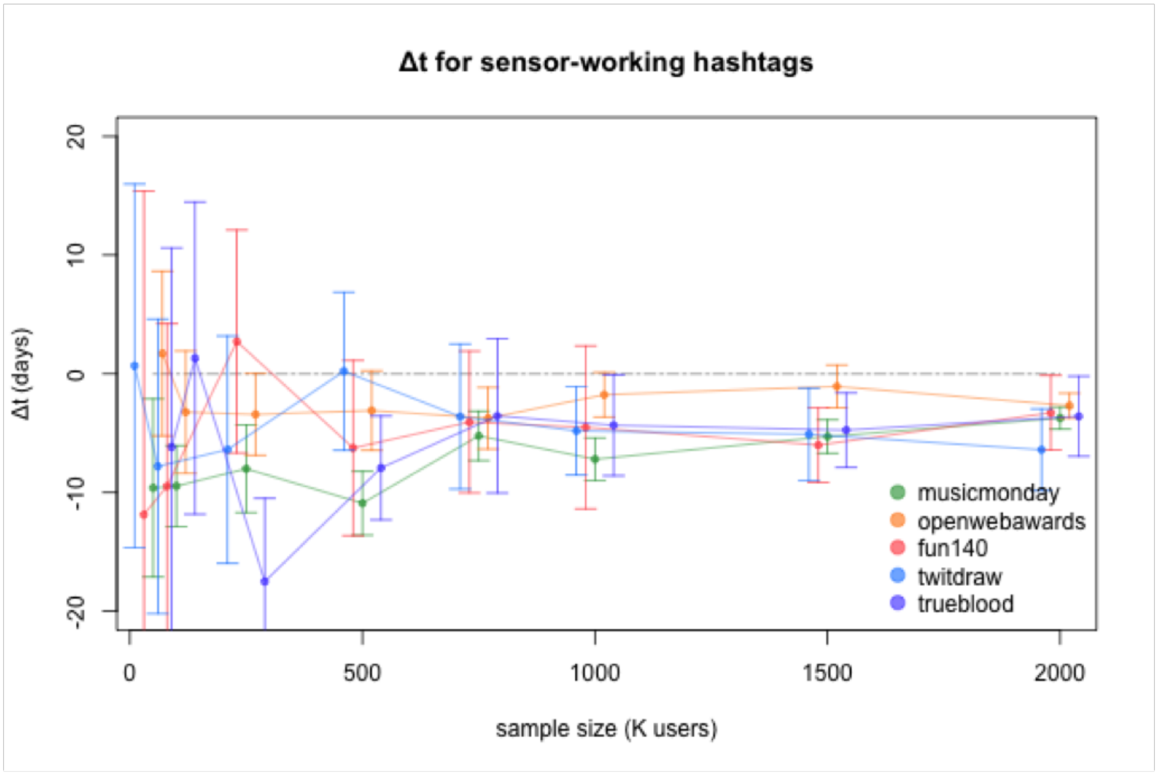

Figure S9. Lead times in the sensor group compared to the control group (Δt^{α}) in different sample sizes for viral hashtags: *#musicmonday*, *#openwebawards*, *#fun140*, *#twitdraw* and *#forasarney*.

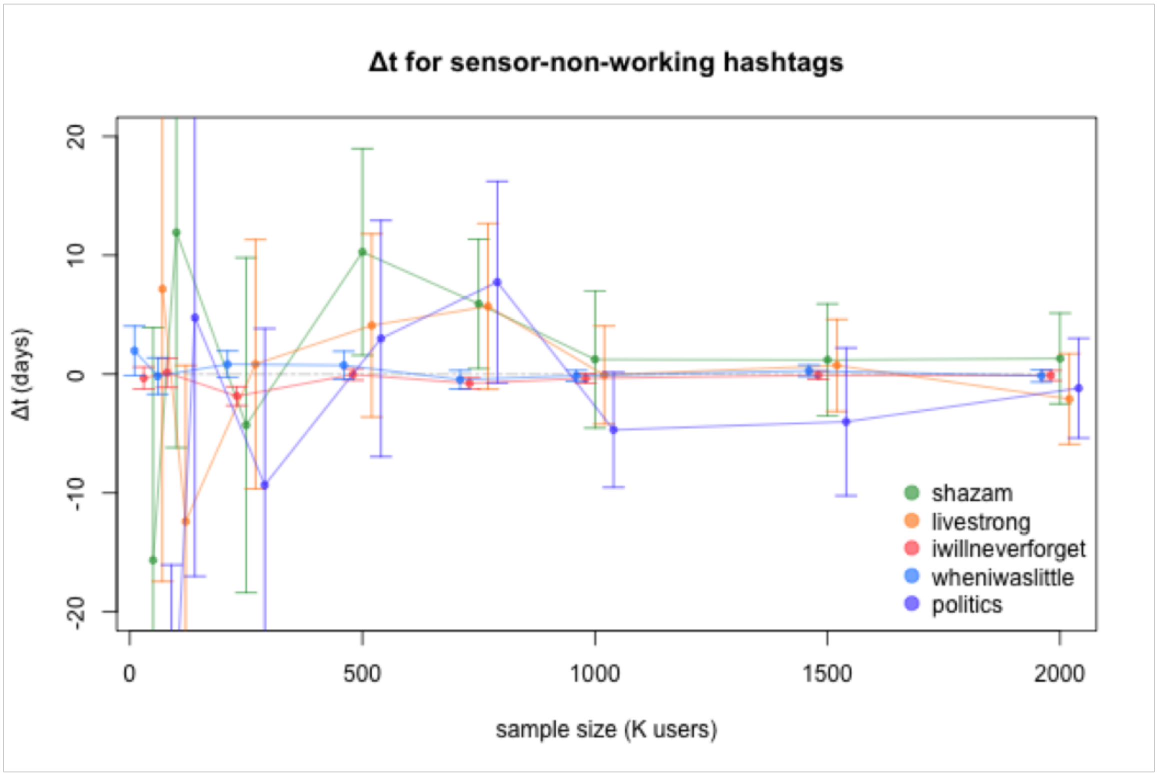

Figure S10. Lead times in the sensor group compared to the control group (Δt^{α}) for nonviral hashtags: *#shazam*, *#livestrong*, *#iwillneverforget*, *#wheniwaslittle* and *#politics*.

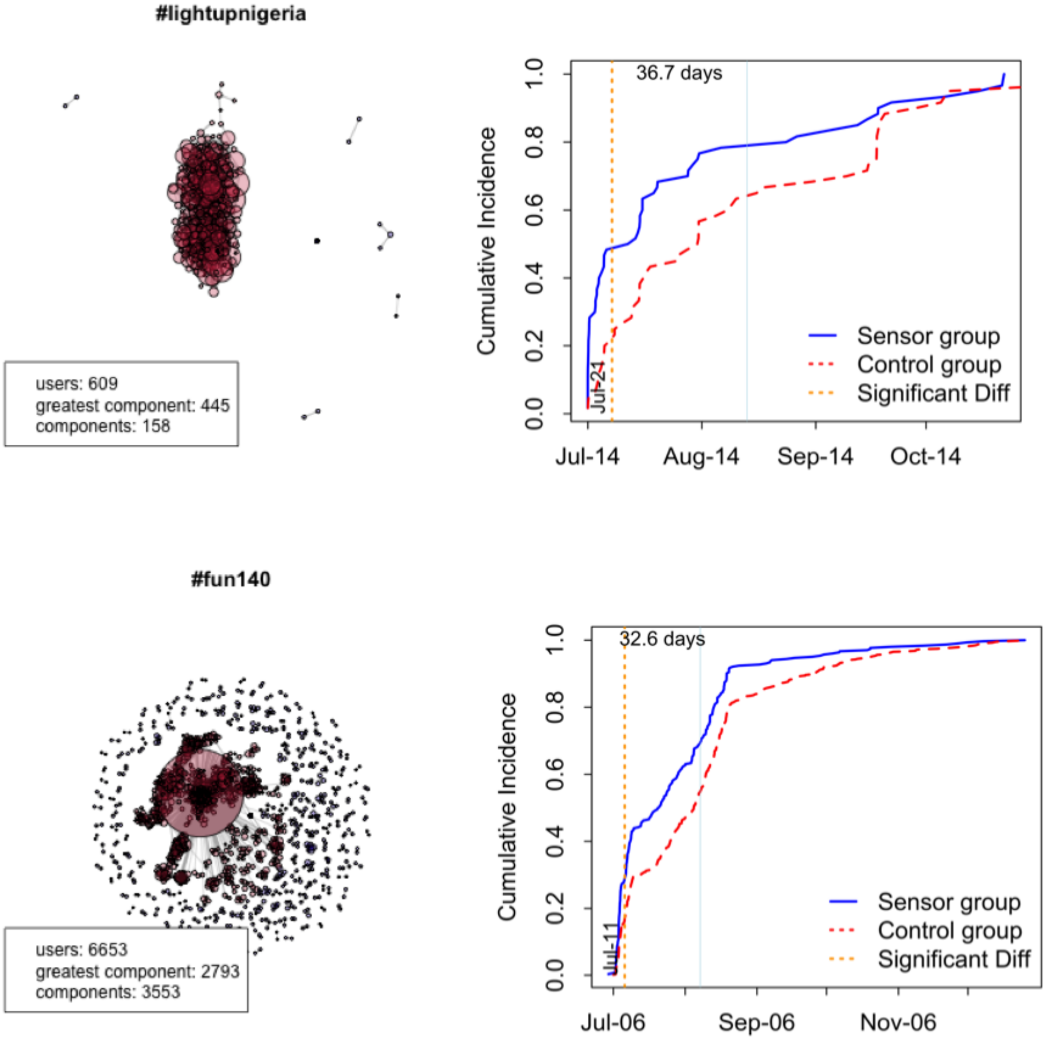

Figure S11. Networks and cumulative distributions for the hashtags showing the biggest lead times in Figure 3 of the main text. *#lightupnigeria* is a small network showing the biggest difference between the cumulative distributions of the control and sensor groups. *#fun140* shows the third biggest lead time of the hashtags analyzed and its network displays an unusually large hub at its center.

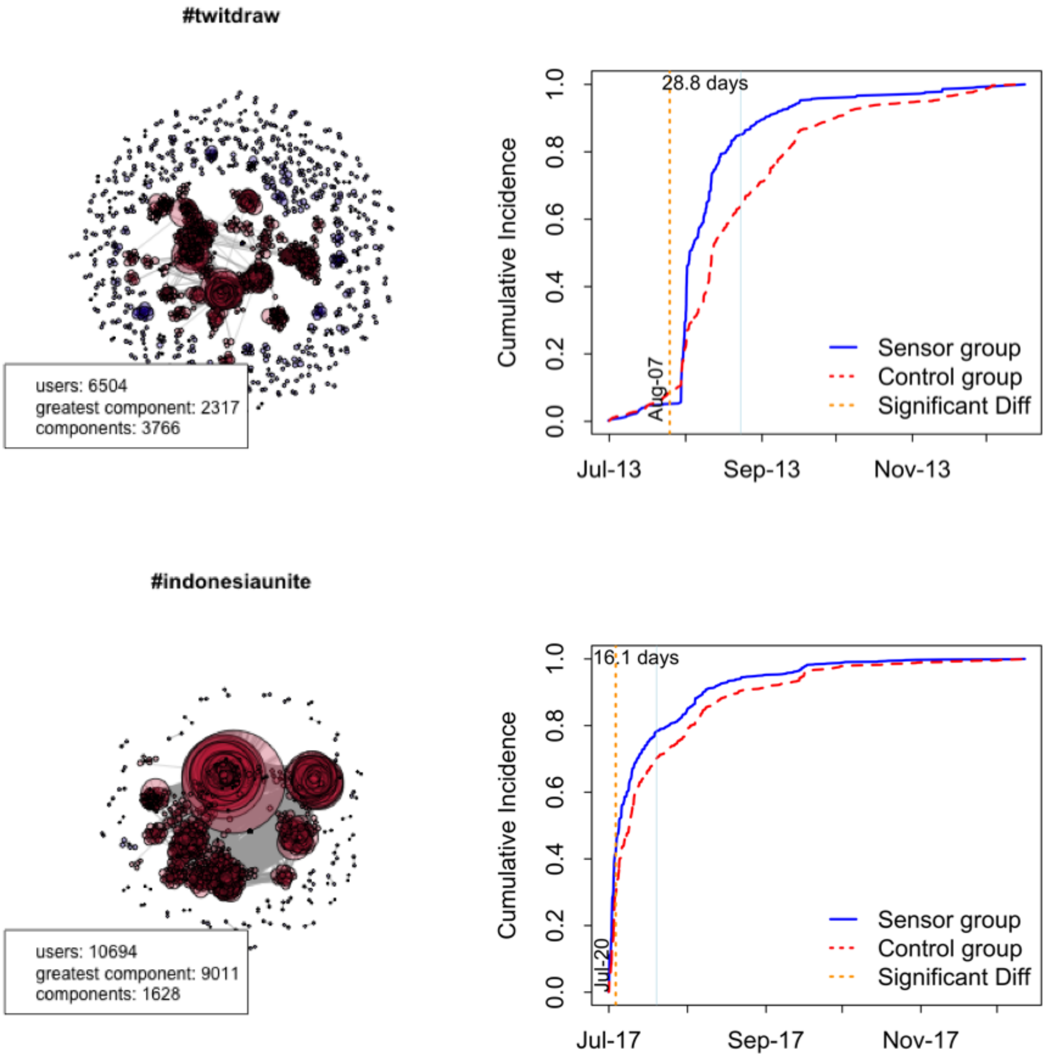

Figure S12. Two additional examples of hashtag networks and cumulative tweets showing a considerable lead time for the sensor group.

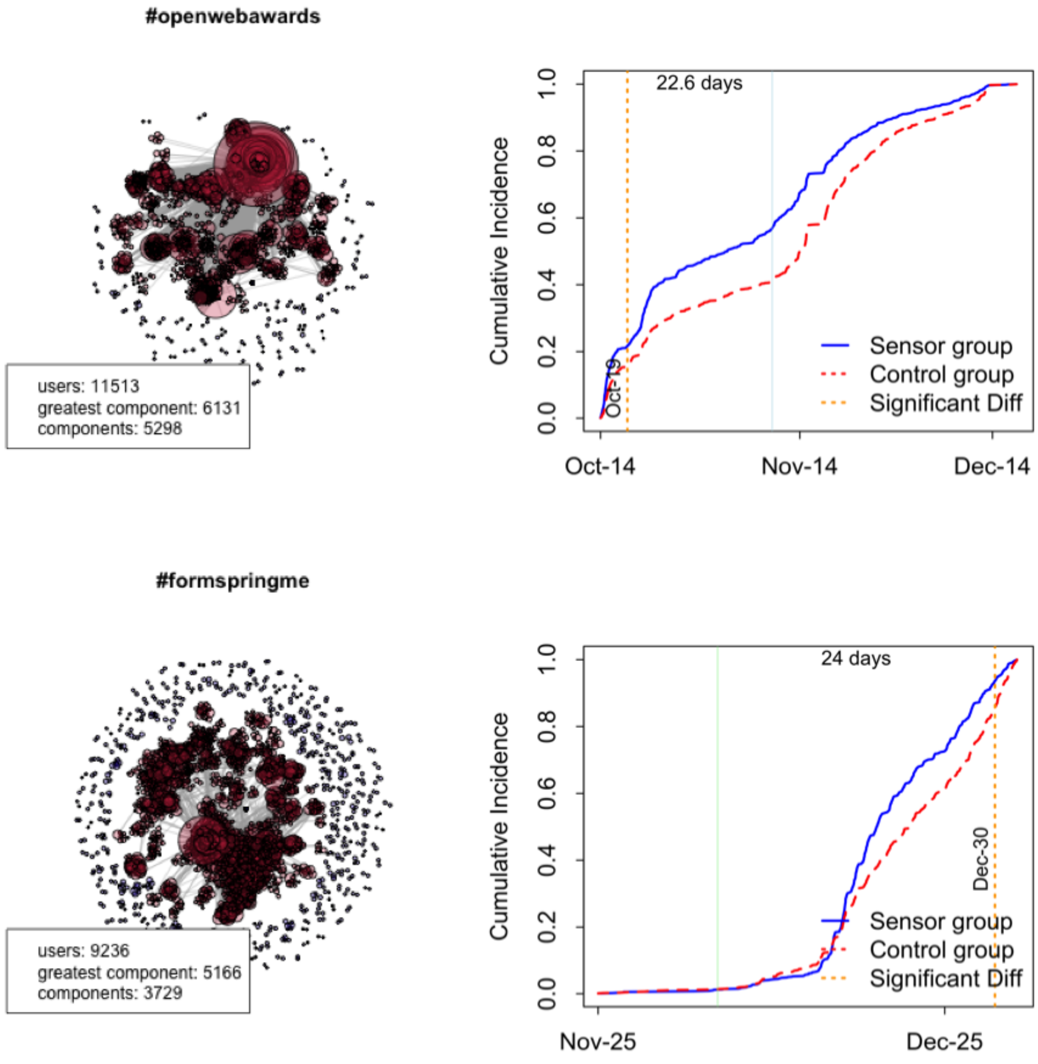

Figure S13. Two additional examples of hashtag networks and cumulative tweets showing a considerable lead time for the sensor group.

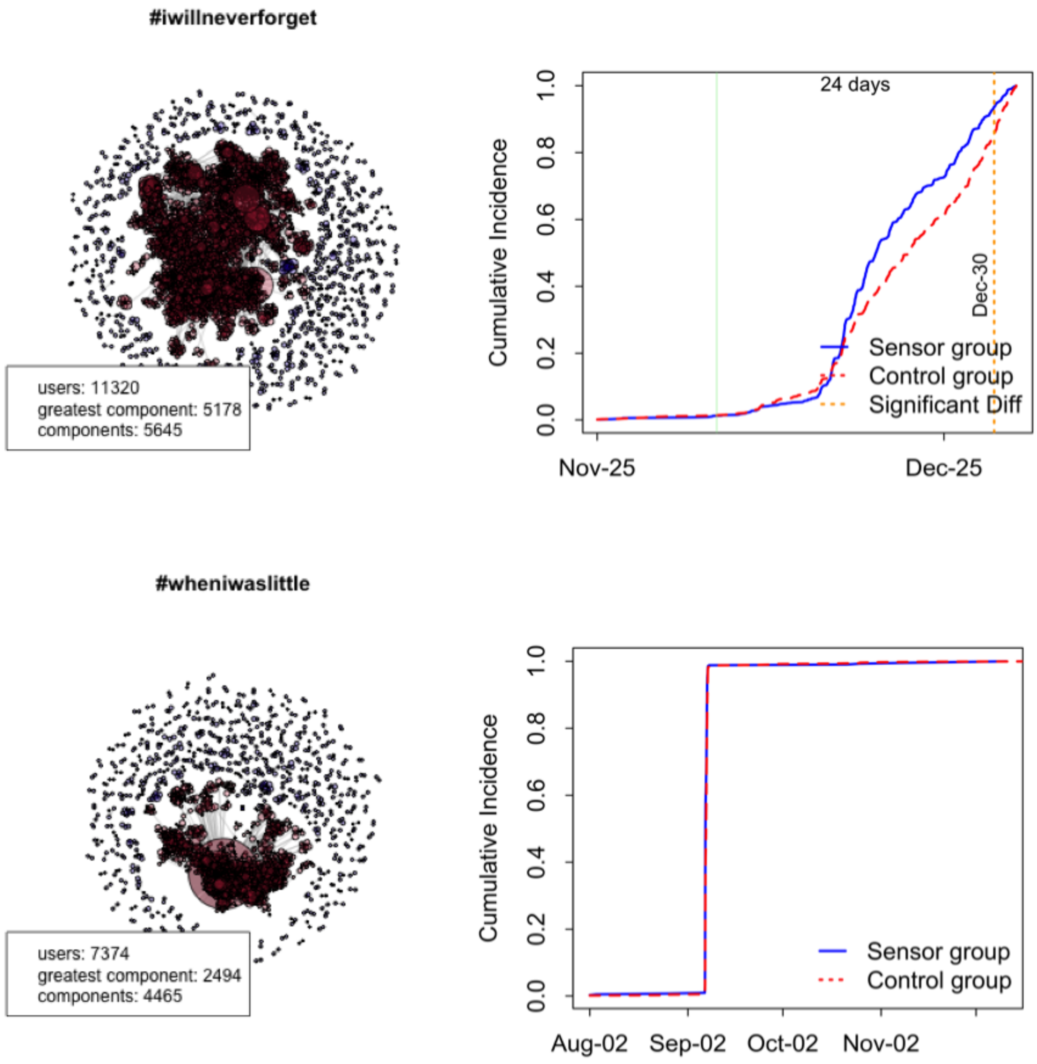

Figure S14. Although these two hashtag networks have large giant components, they show signs of very rapid increase in usage, suggesting the sensor mechanism may not give much advance warning in these cases. Their lead times overlap with those based on randomly shuffling the data as shown in Figure 3 in the main text.

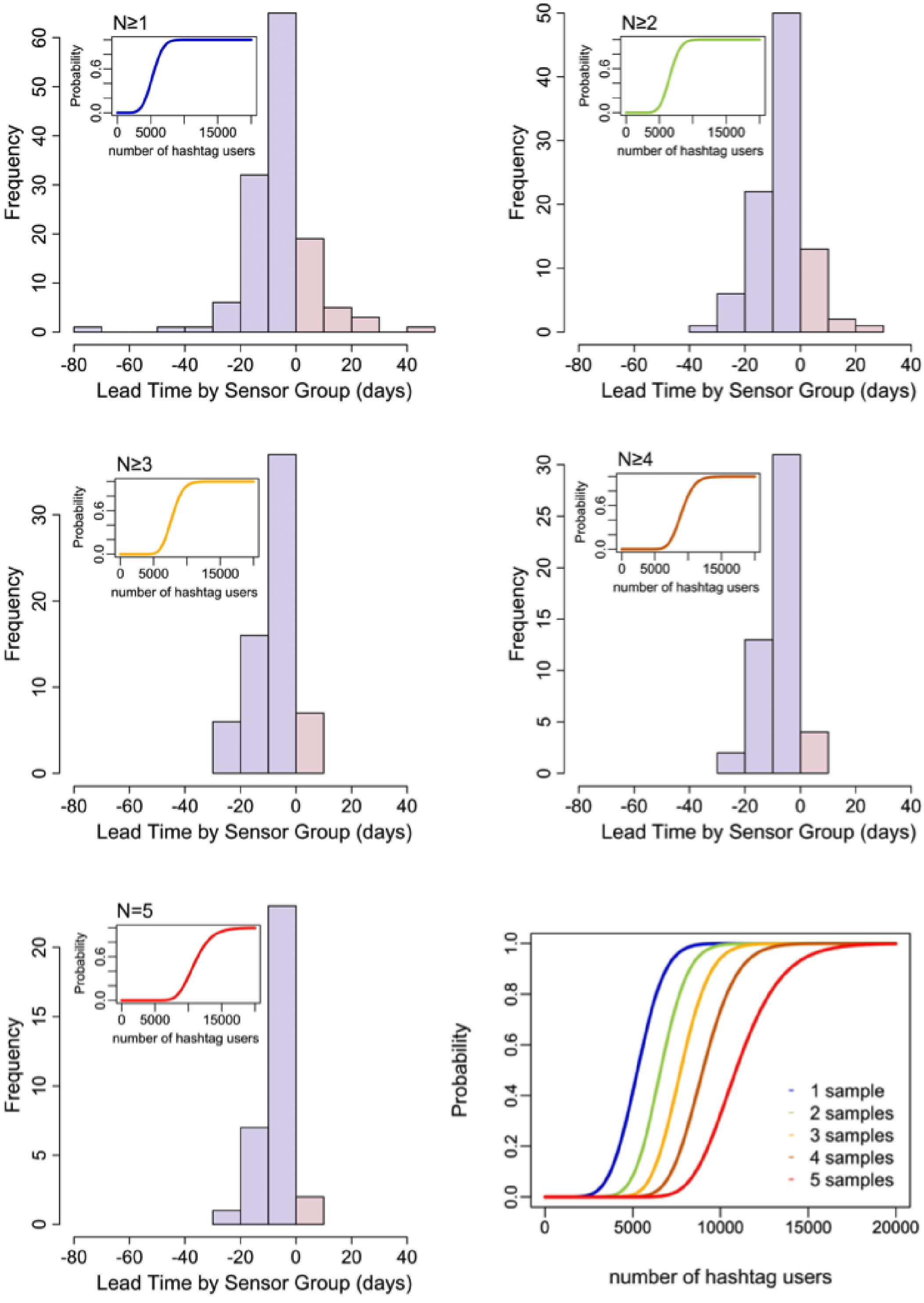

Figure S15. Sensor lead time (Δt^{α}) distributions, total number of users, and probability distributions for hashtags having at least 10 users in 1 or more of 5 random samples of 50,000 users, 2 or more, 3 or more, 4 or more, and 5 or more.

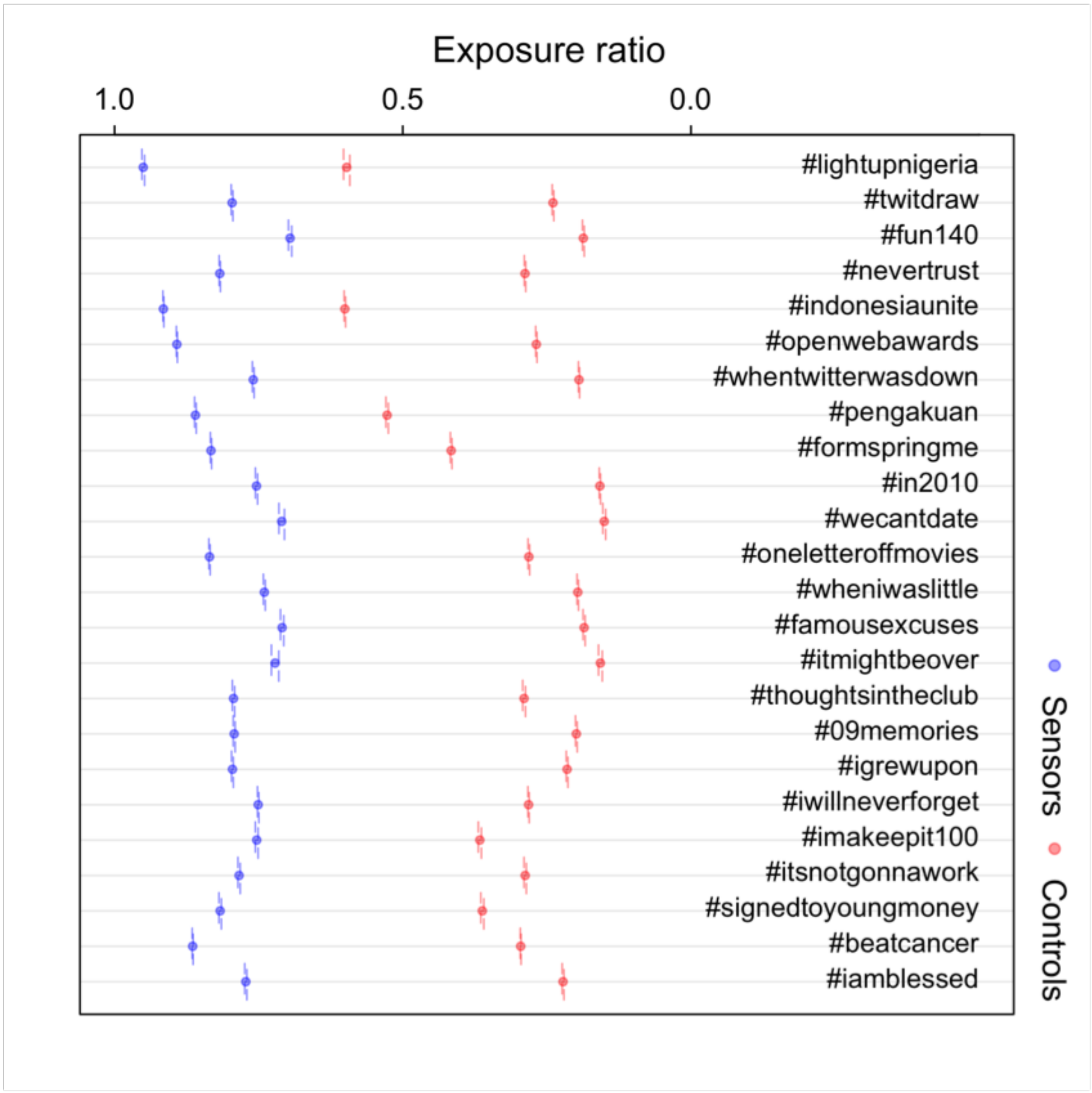

Figure S16. Exposure rate of sensor and control populations. Although they use hashtags earlier than controls on average (as shown in Figure 3 of the main text), sensors have higher exposure rates than controls, meaning that they are better at transmitting information in Twitter than they are at introducing new information from other sources. Controls, on the other hand, seem to use more hashtags to which they have not been previously exposed in Twitter.

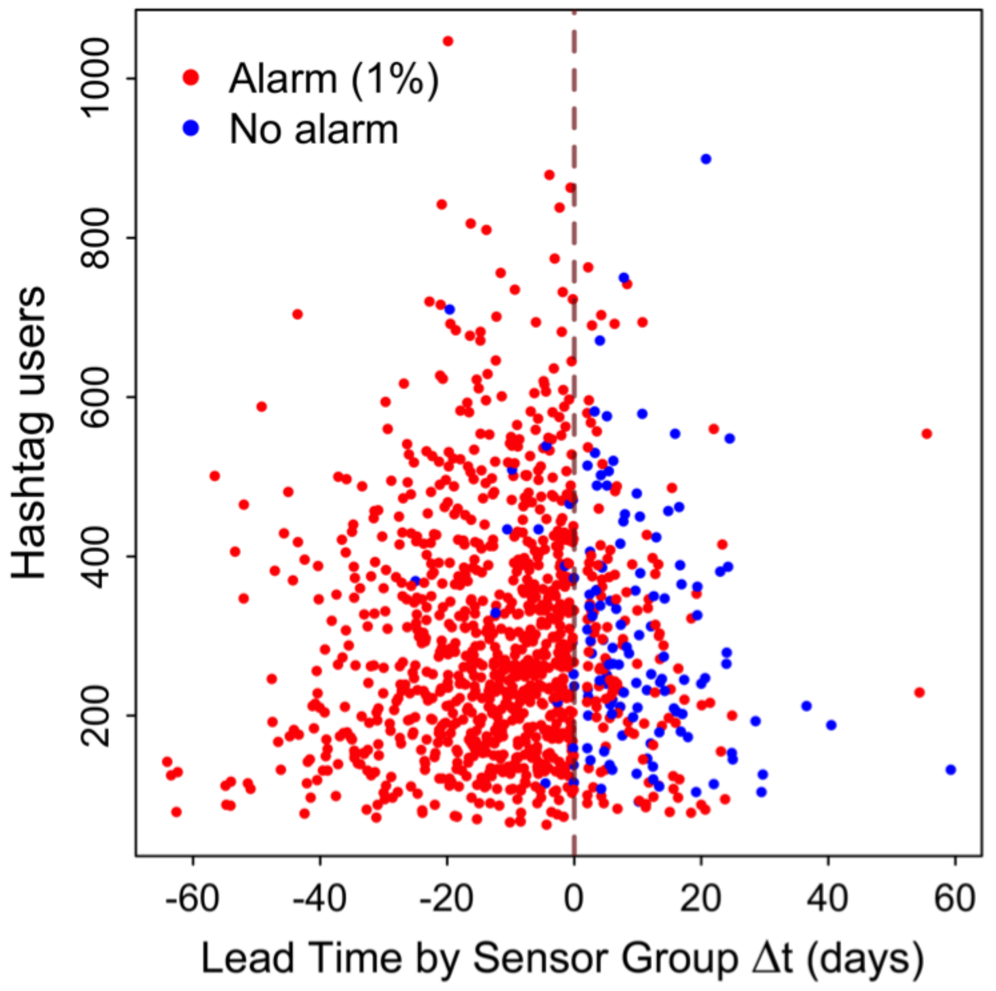

Figure S17. Sensor lead time versus total number of hashtag users for 2,000 hashtags ranked from 4,000 to 6,000 by total usage in the last 6 months of 2009. We restricted analysis to cases where at least 10 users in the control sample follows at least another 5 hashtag users (1,111 hashtags out of the 2,000 meet this requirement). Red dots indicate hashtags that set off a divergence alarm (the sensor distribution was greater than the control distribution at some point in time by 1%) and blue dots indicate hashtags that did not achieve this divergence. These results show that the most used hashtags tend to have lead times close to zero, and hashtags that show an early divergence tend also to have a large average lead time in the sensor group compared to the control over the full course of the epidemic.

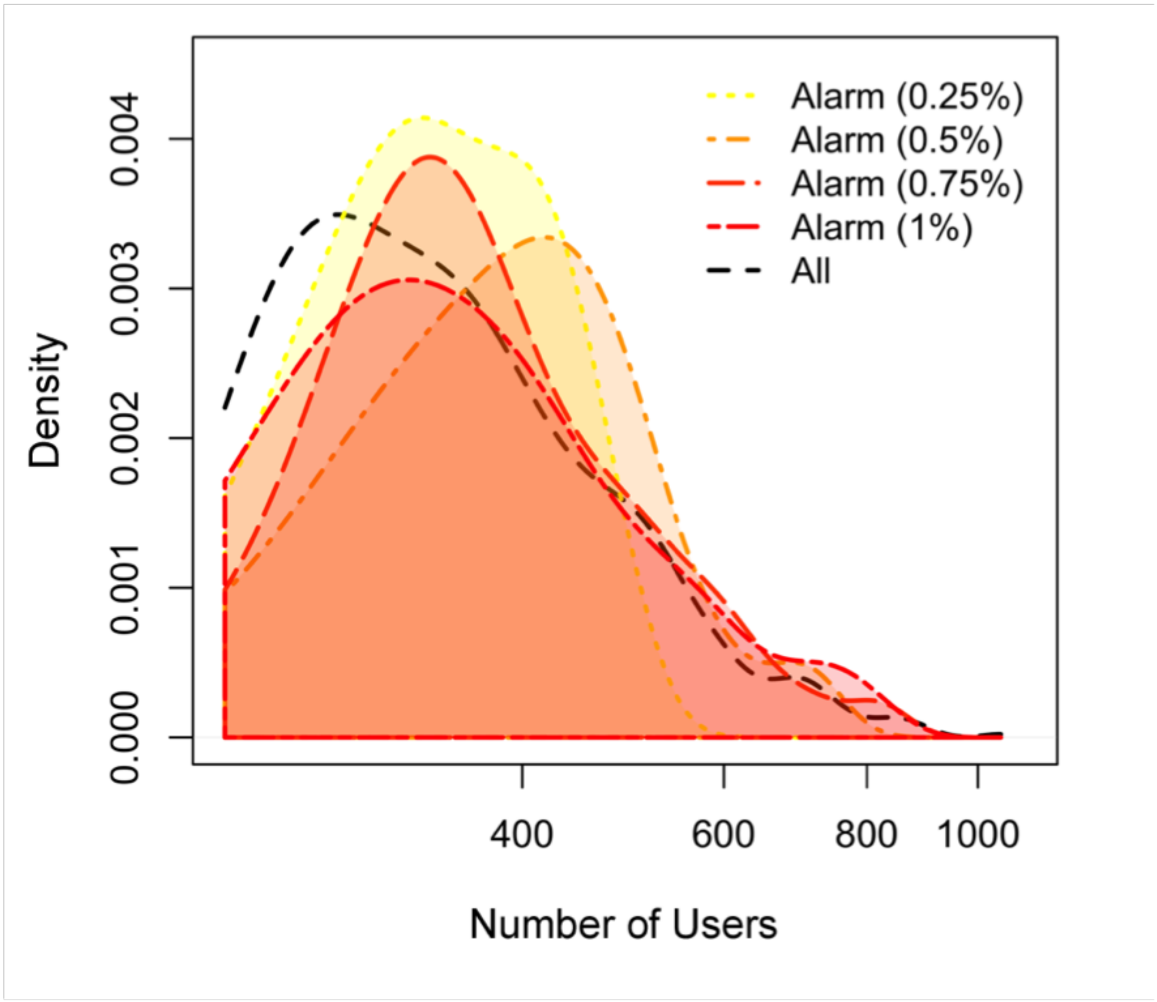

Figure S18. Distribution of number of hashtag users for hashtags that trigger a divergence alarm (under different requirements for the size of the divergence) compared to those that do not set off an alarm (using the same sample as described in Supplementary Figure S17). A divergence alarm is triggered when there is X% more of the sensor population using a hashtag than the control population, where X is 0.25% (yellow), 0.5 % (orange), 0.75% (red-orange) or 1% (red). The best performance occurs for intermediate percentages because some highly viral processes proceed too quickly to detect (as shown by the higher rates of usage clustered near lead times of zero in Supplementary Figure S17).

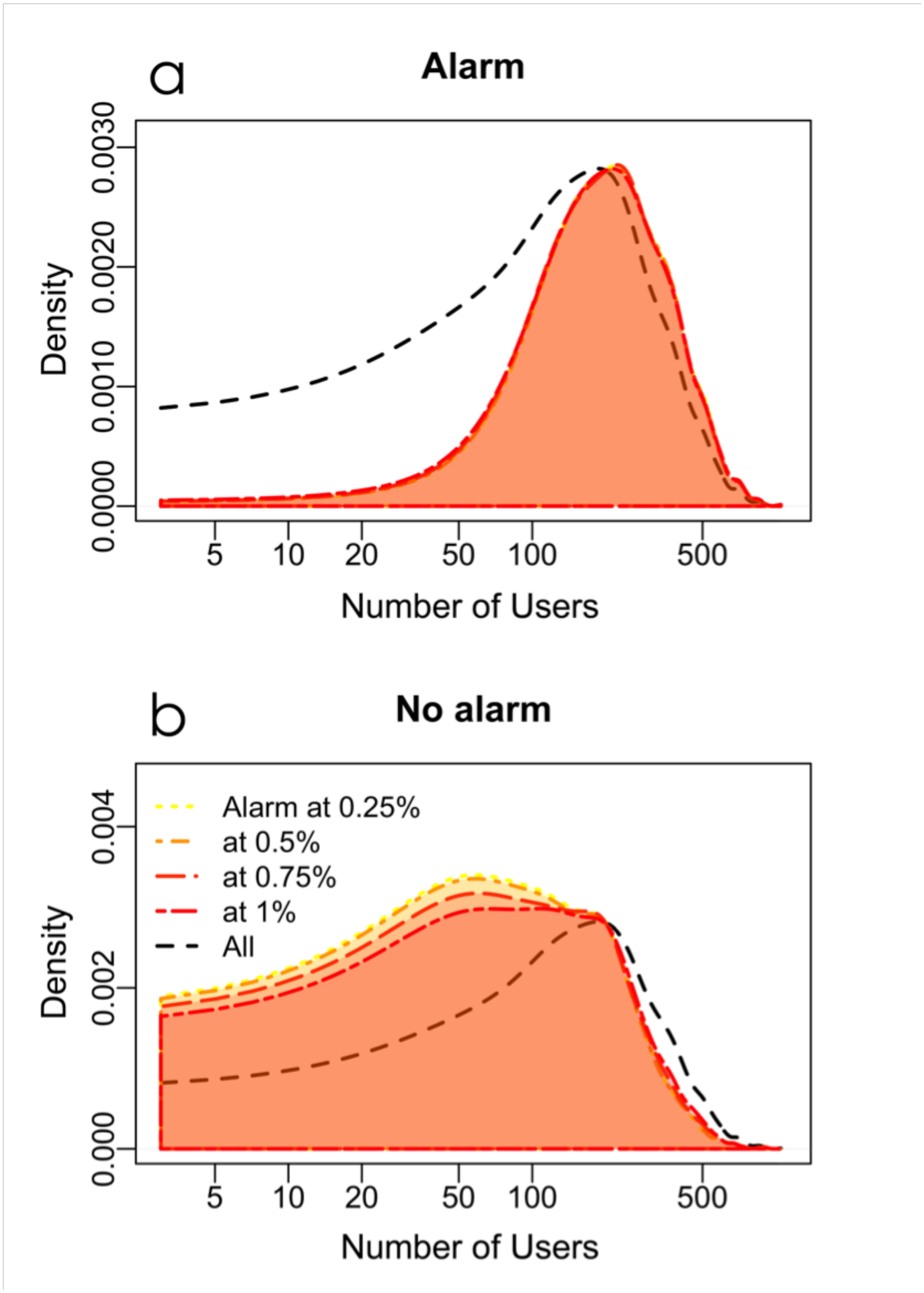

Figure S19. Distribution of number of users for hashtags triggering a divergence alarm vs. not triggering an alarm using different alarm thresholds (based on the same sample as described in Supplementary Figure S17).

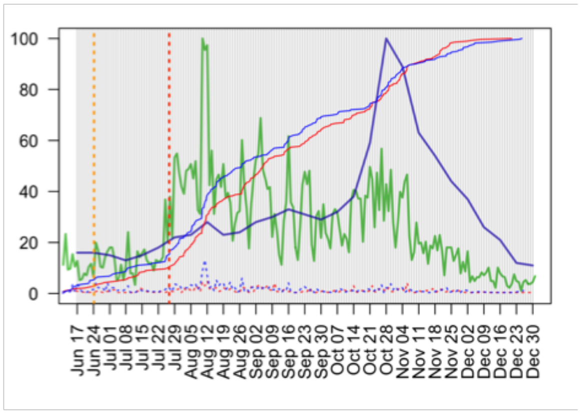

A) *#H1N1* vs. "H1N1". influenza pandemic, and the second of the two pandemics involving H1N1 influenza virus (after 1918 flu pandemic).

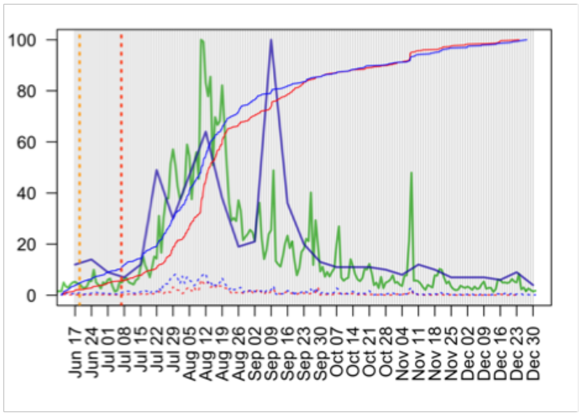

B) *#Healthcare* vs "healthcare obama". Public attention on Obamas Health Care reform.

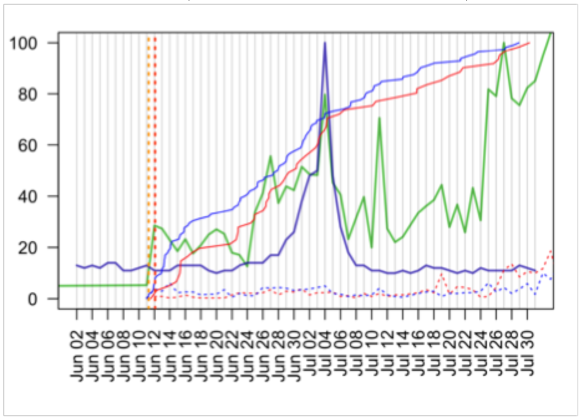

C) *#TeaParty* vs. "tea party". Tea Party protests were held the weekend of July 4, 2009, coinciding with American Independence Day.

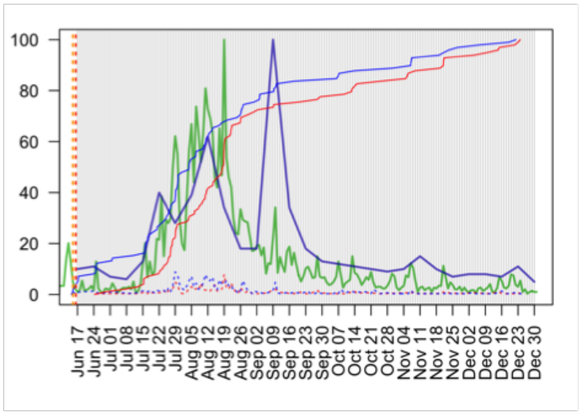

D) *#ObamaCare* vs. "obama care". Public attention on what will become the Affordable Health Care for America Act.

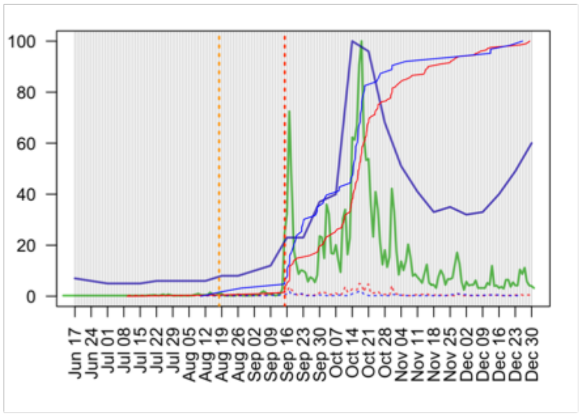

E) *#Uncharted2* vs. "uncharted 2". PlayStation 3 video game released in October 2009.

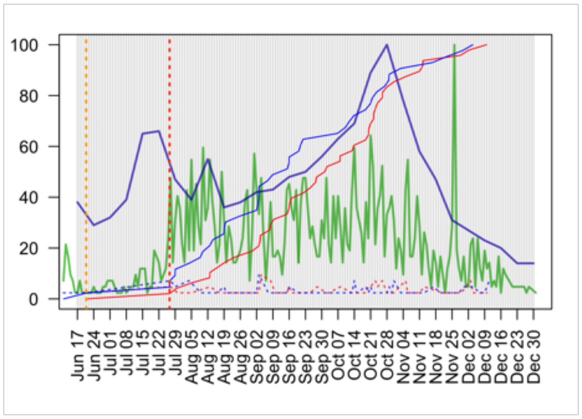

F) *#Flu* vs "flu".

Figure S20. Twitter hashtags vs. Google searches. Dashed green line: Twitter daily incidence. Purple line: Google daily incidence. Blue line: Sensor/Control cumulative incidence: blue/red line. Sensor/Control daily incidence: blu/red dotted line. Orange/Red vertical dashed lines: divergence alarms on 2.5%/5% divergences

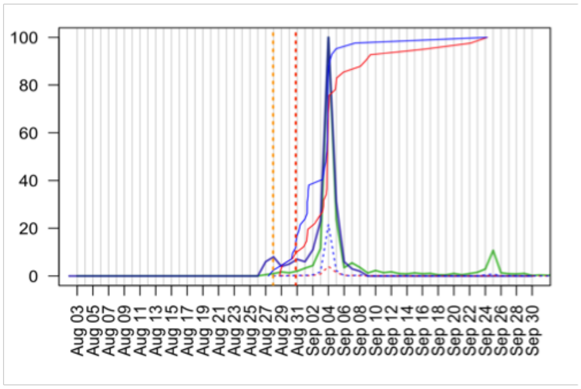

G) *#NoMasChavez* vs. "no mas chavez". September 4th youth Colombian democratic Manifesto against Chavezs government.

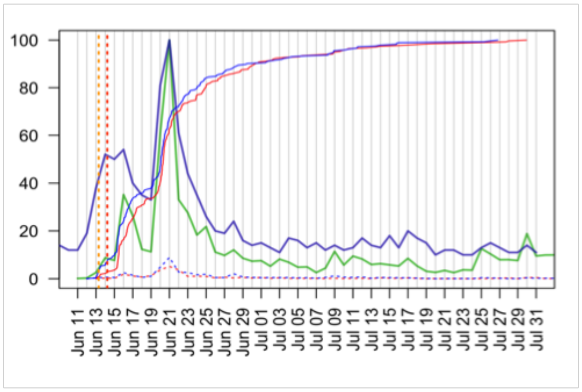

H) *#Tehran* vs. "Tehran". Protests Flare in Tehran as Opposition Disputes Vote (New York Times) June 13th 2009.

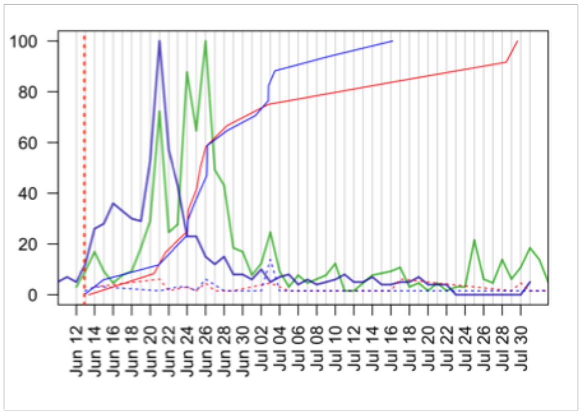

I) *#Revolution* vs. "Iran revolution". Protests against the disputed victory of Iranian President Mahmoud Ahmadinejad starting 13 June 2009.

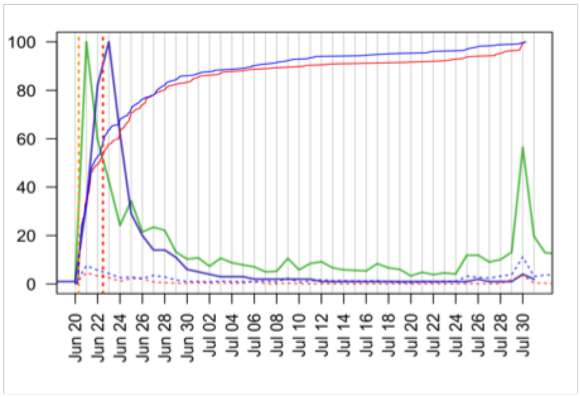

J) *#Neda* vs "Neda". 20th June 2009, Neda Agha-Soltan is shot during Iran election protests.

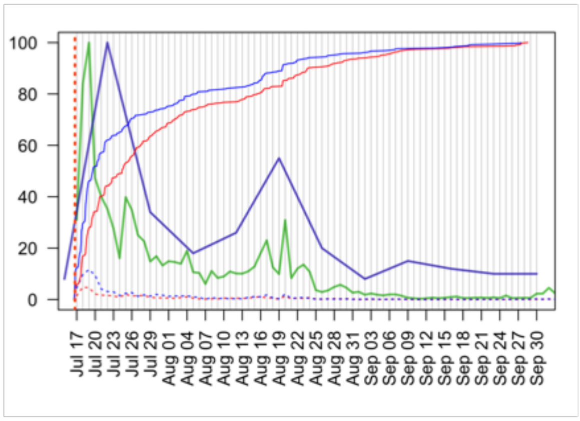

K) *#IndonesiaUnite* vs. "Indonesia unite". 17 July 2009, the JW Marriott and Ritz-Carlton Hotels in Jakarta, Indonesia, are hit by separate bombings.

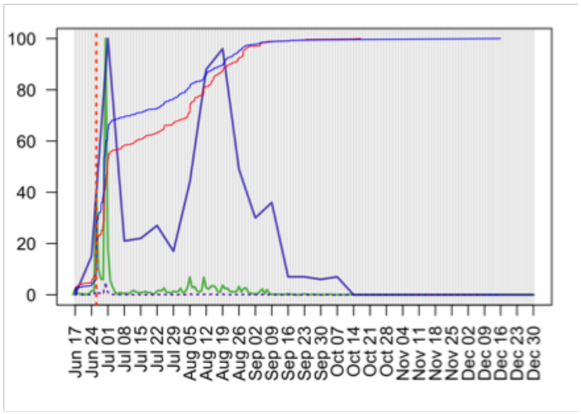

L) *#ForaSarney* vs. "fora Sarney". Public movement against Jose Sarney, president of the Brazilian Senate, after corruption accusations.

Figure S20. Continued. Twitter hashtags vs. Google searches.

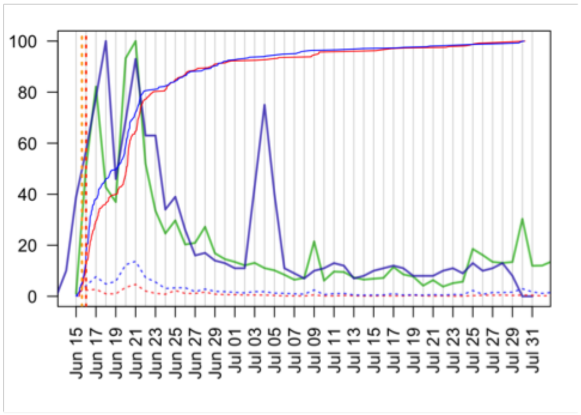

M) *#Gr88* vs. "Iran green". Iranian Green Revolution. Protests began the night of 12 June 2009, following the announcement that incumbent President Mahmood Ahmadinejad had won nearly 60 percent despite several reported irregularities.

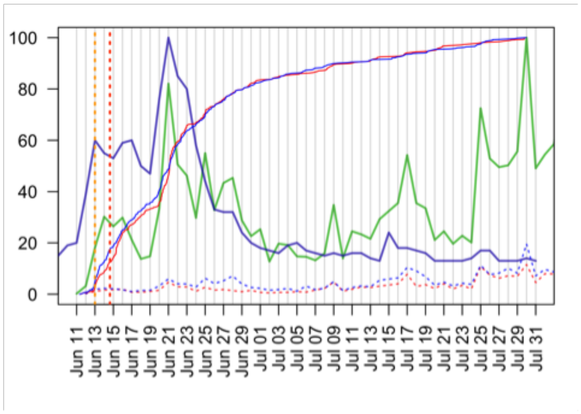

N) *#Iran* vs. "Iran". Protests following the 2009 Iranian presidential election against the disputed victory of Iranian President Mahmoud Ahmadinejad and in support of opposition candidates Mir-Hossein Mousavi and Mehdi Karroubi occurring in major cities in Iran and around the world starting 13 June 2009.

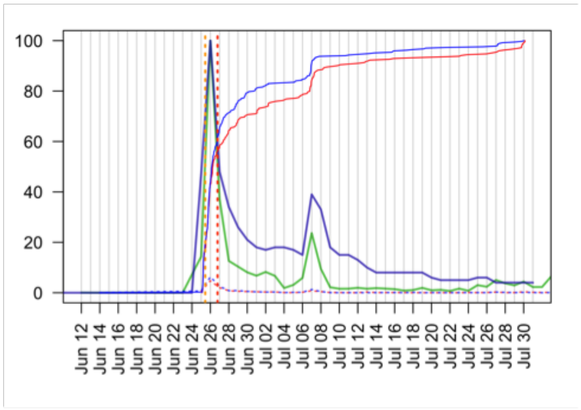

O) *#MichaelJackson* vs "Michael Jackson". Michael Jackson dies of acute propofol and benzodiazepine intoxication on June 25, 2009, after suffering from cardiac arrest.

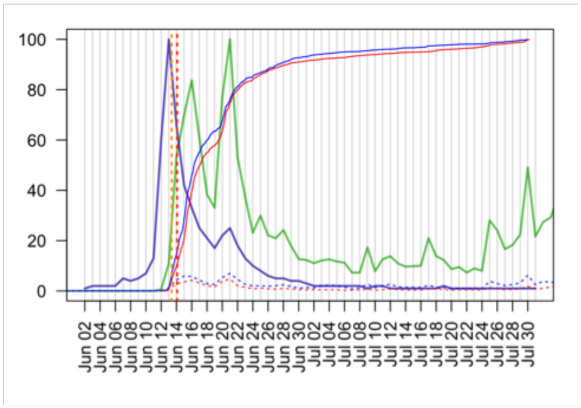

P) *#IranElection* vs "Iran election". Iran's tenth presidential election was held on 12 June 2009. Al Jazeera English described the 13 June situation as the "biggest unrest since the 1979 revolution". On 15 June, Mousavi rallied, with anywhere from hundreds of thousands to three million, of his supporters in Tehran. Competing rallies for Mousavi and for Ahmadinejad took place on 16 June. Reports from the state media and elsewhere stated on 16 June that seven people have died in all of the protests so far.

Figure S20. Continued. Twitter hashtags vs. Google searches.

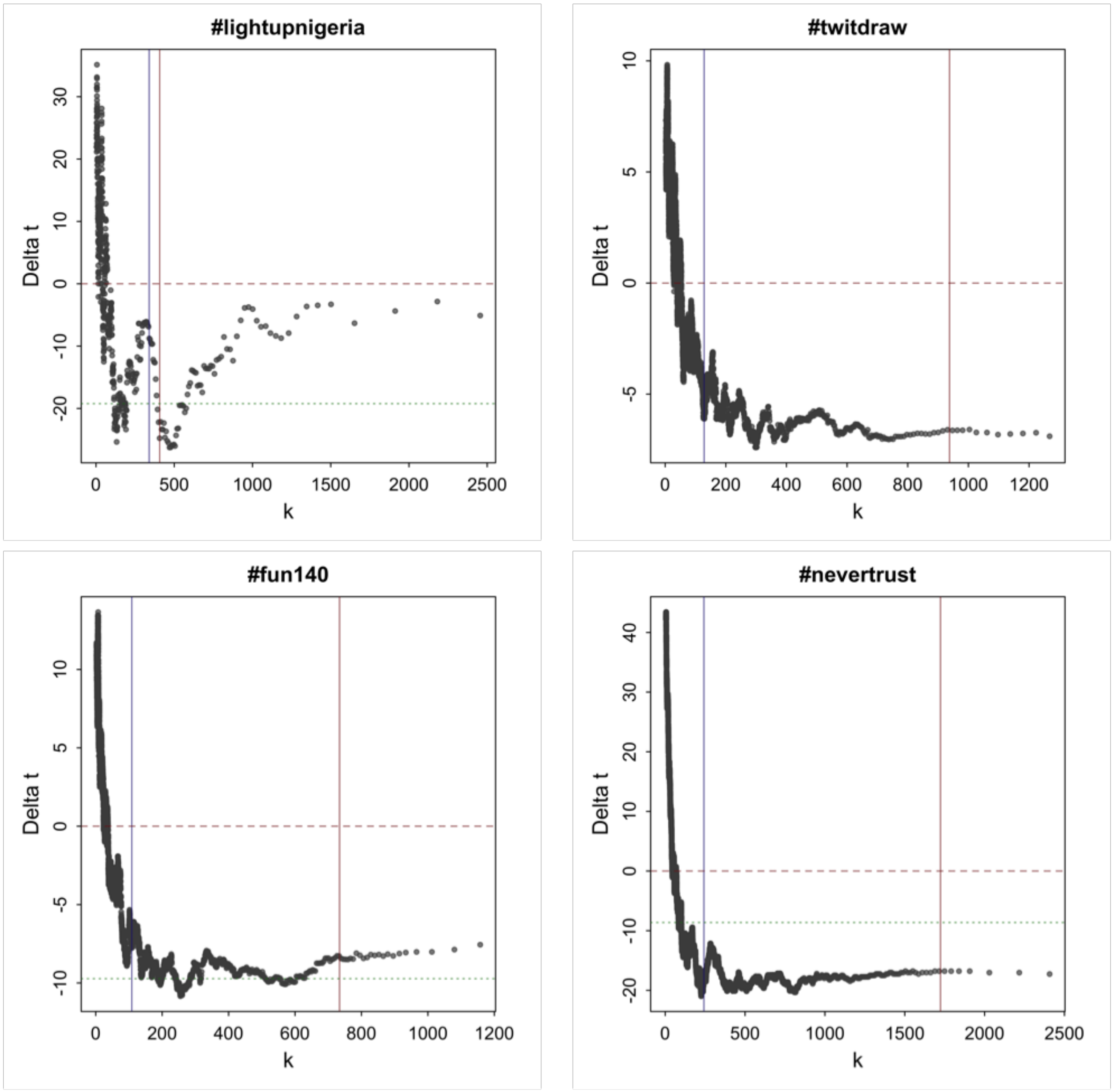

Figure S21. Lead time Δt for sensor groups of various mean degree k compared to control groups. Horizontal green dotted line: Δt obtained using friends as sensors.

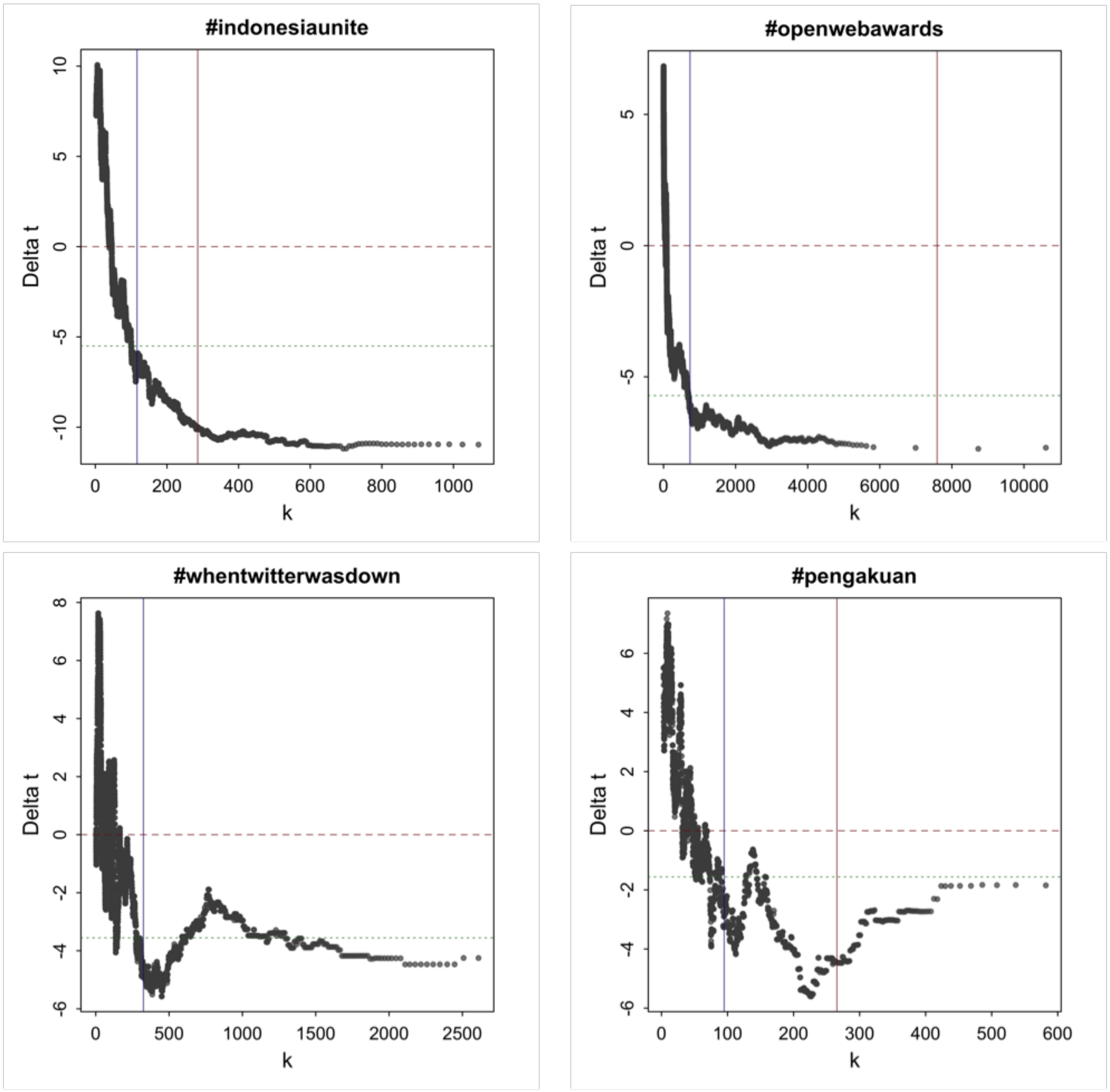

Figure S21. Continued. Lead time Δt for sensor groups of various mean degree k compared to control groups.

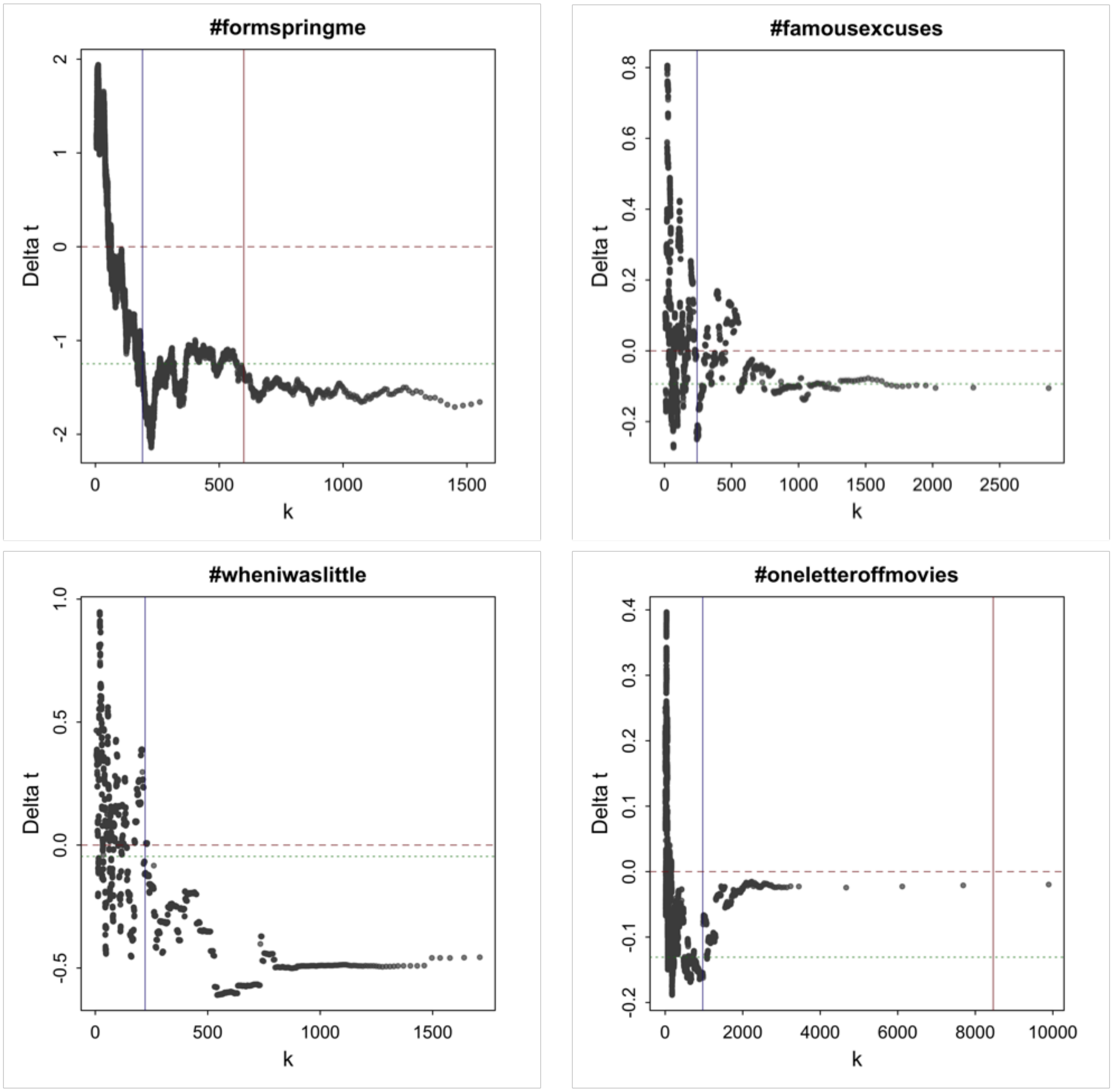

Figure S21. Continued. Lead time Δt for sensor groups of various mean degree k compared to control groups.

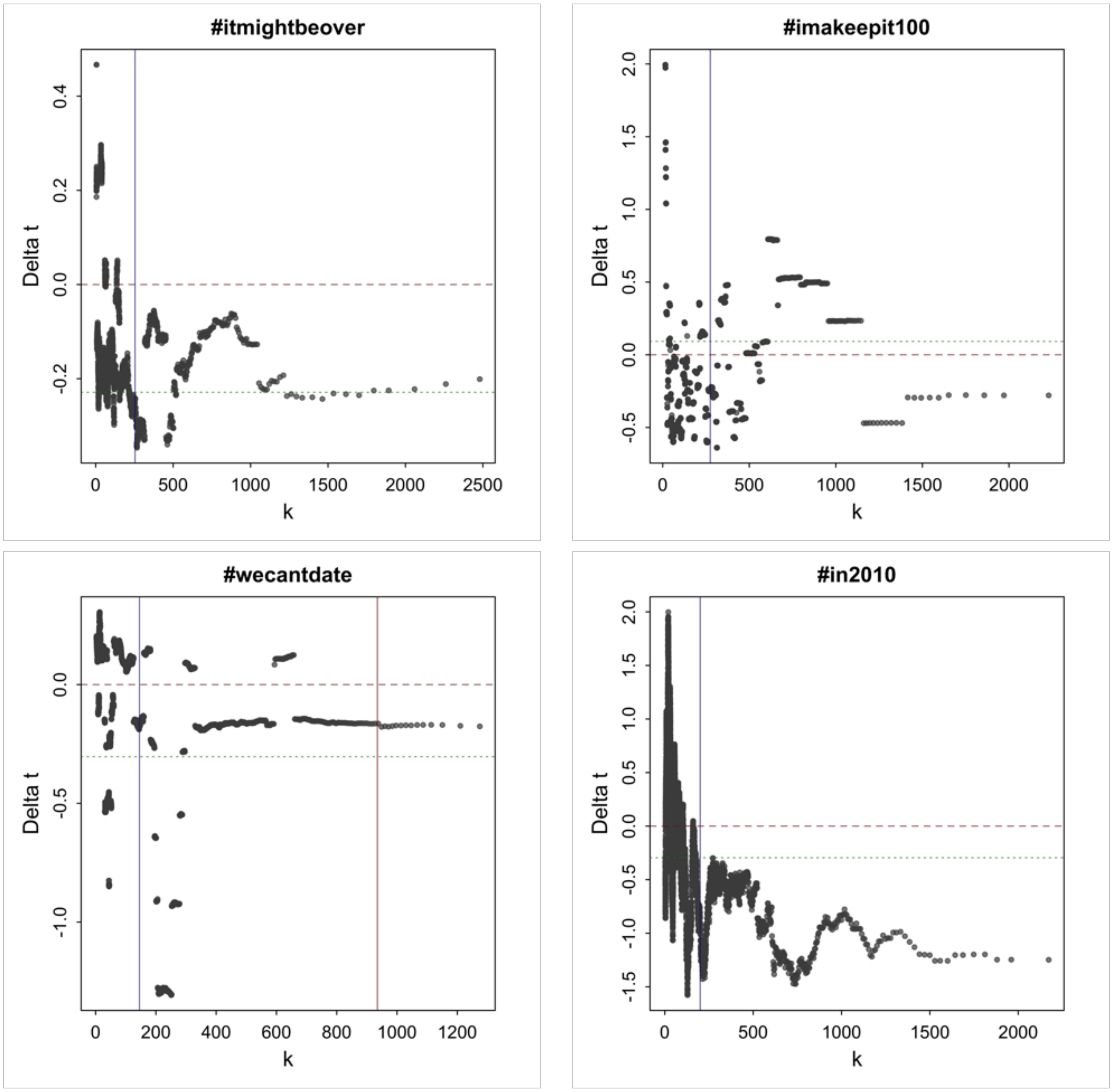

Figure S21. Continued. Lead time Δt for sensor groups of various mean degree k compared to control groups.

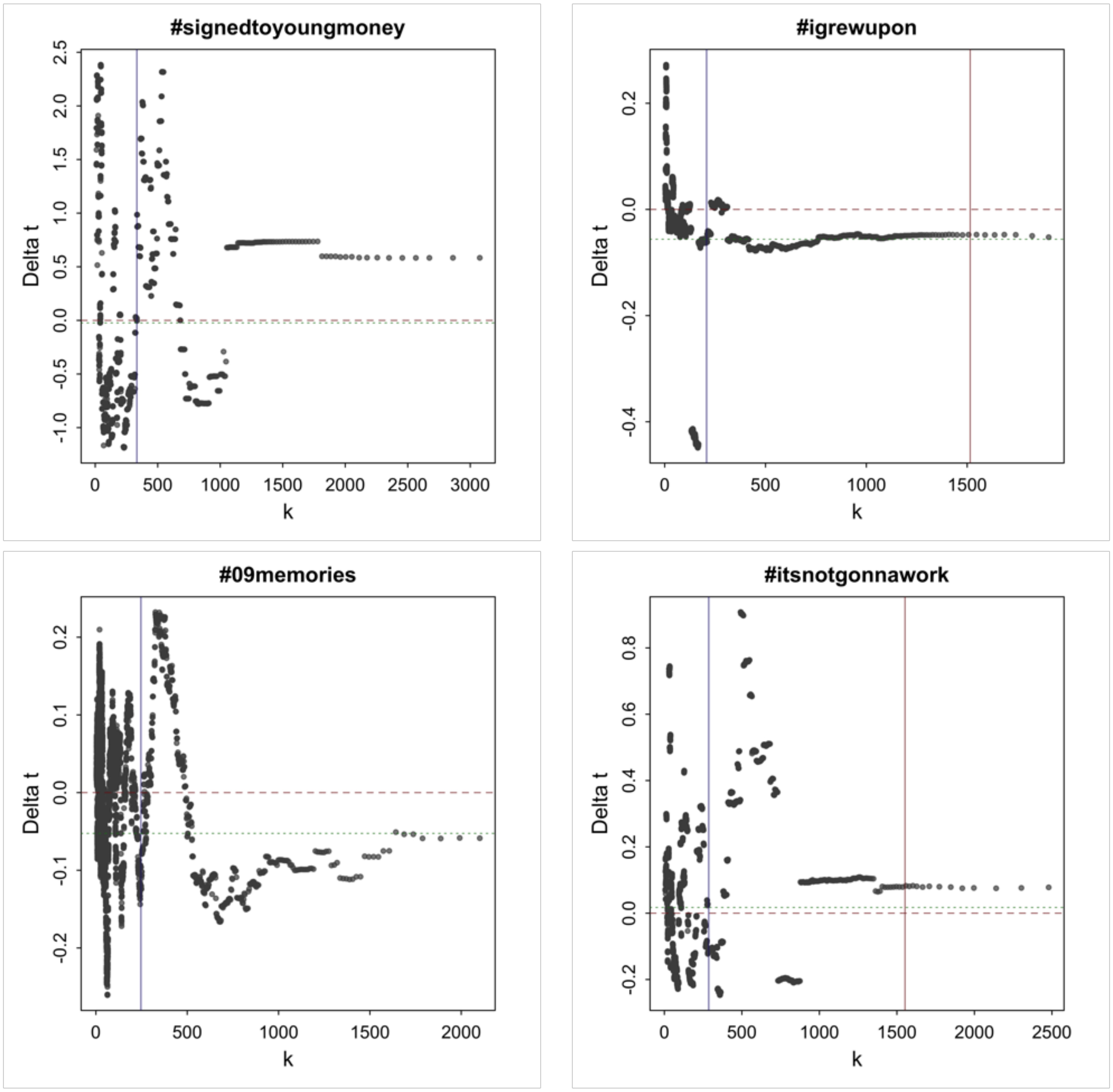

Figure S21. Continued. Lead time Δt for sensor groups of various mean degree k compared to control groups.

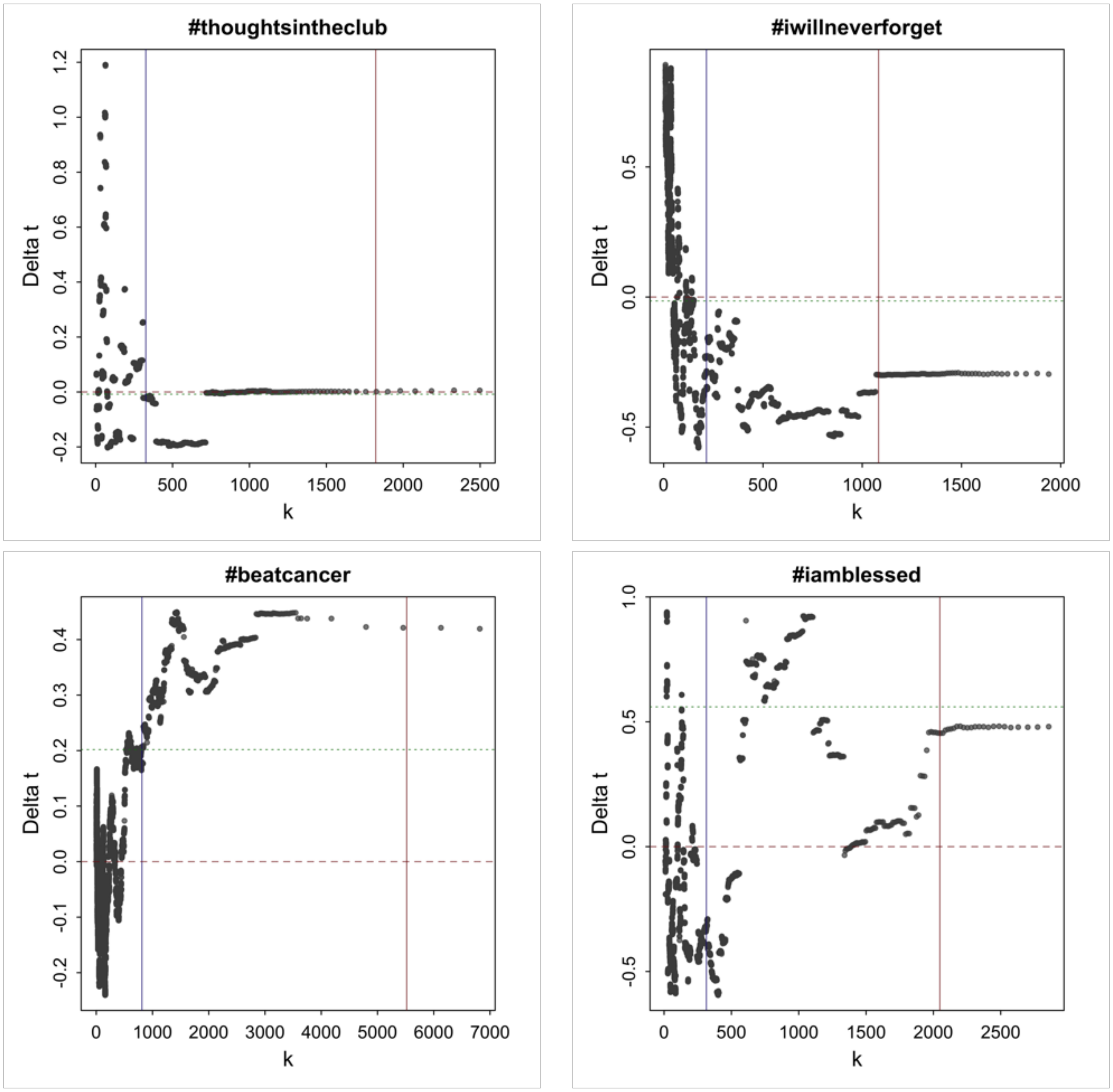

Figure S21. Continued. Lead time Δt for sensor groups of various mean degree k compared to control groups.

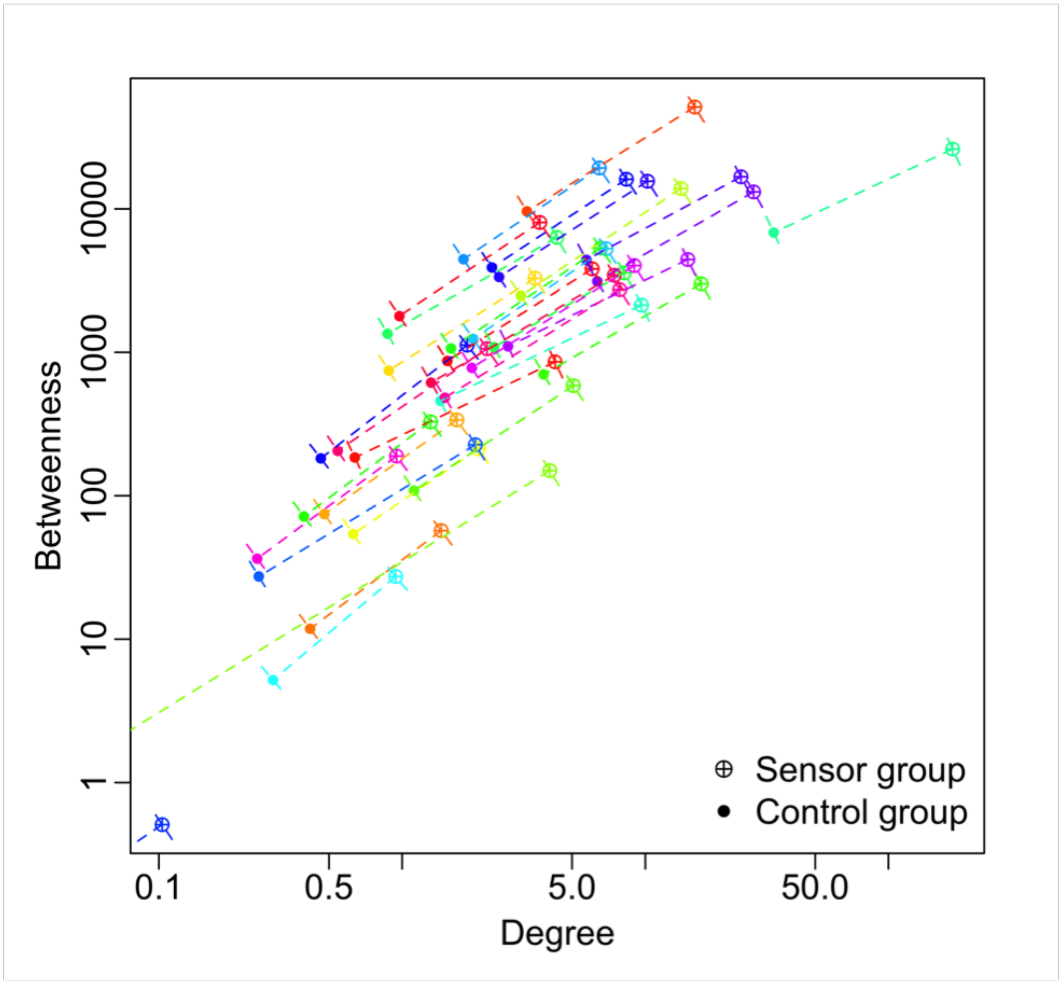

Figure S22. Mean degree and betweenness for 340 control groups and their corresponding sensors of 34 different hashtags user networks (those in Supplementary Figure S3). Each point in the plot corresponds to the mean value of 10 different samples of a single hashtag network. Each hashtag network is drawn in a different color. Control and sensor samples are 10% the size of the user network. The results show the sensor groups have both higher mean degree and higher betweenness than a randomly-chosen control group.
